# Peroxin Pex14/17 Is Required for Trap Formation, and Plays Pleiotropic Roles in Mycelial Development, Stress Response, and Secondary Metabolism in Arthrobotrys oligospora

**DOI:** 10.1128/msphere.00012-23

**Published:** 2023-02-14

**Authors:** Qianqian Liu, Dongni Li, Na Bai, Yingmei Zhu, Jinkui Yang

**Affiliations:** a State Key Laboratory for Conservation and Utilization of Bio-Resources in Yunnan, Key Laboratory for Southwest Microbial Diversity of the Ministry of Education, Yunnan University, Kunming, China; b School of Life Science, Yunnan University, Kunming, China; University of Georgia

**Keywords:** *Arthrobotrys oligospora*, peroxin, conidiation, trap formation, lipid droplet, reactive oxygen species, mycelial growth and development, peroxisome biogenesis

## Abstract

The peroxins encoded by *PEX* genes involved in peroxisome biogenesis play a crucial role in cellular metabolism and pathogenicity in fungi. Herein, we characterized a filamentous fungus-specific peroxin Pex14/17 in the Arthrobotrys oligospora, a representative species of nematode-trapping fungi. The deletion of *AoPEX14/17* resulted in a remarkable reduction in mycelial growth, conidia yield, trap formation, and pathogenicity. Compared with the wild-type strain, the Δ*Aopex14/17* mutant exhibited more lipid droplet and reactive oxygen species accumulation accompanied with a significant decrease in fatty acid utilization and tolerance to oxidative stress. Transcriptomic analysis indicated that *AoPEX14/17* was involved in the regulation of metabolism, genetic information processing, environmental information processing, and cellular processes. In subcellular morphology, the deletion of *AoPEX14/17* resulted in a decrease in the number of cell nuclei, autophagosomes, and Woronin bodies. Metabolic profile analysis showed that AoPex14/17 affects the biosynthesis of secondary metabolites. Yeast two-hybrid assay revealed that AoPex14/17 interacted with AoPex14 but not with AoPex13. Taken together, our results suggest that Pex14/17 is the main factor for modulating growth, development, and pathogenicity in *A. oligospora*.

**IMPORTANCE** Peroxisome biogenesis genes (*PEX*) play an important role in growth, development, and pathogenicity in pathogenic fungi. However, the roles of *PEX* genes remain largely unknown in nematode-trapping (NT) fungi. Here, we provide direct evidence that AoPex14/17 regulates mycelial growth, conidiation, trap formation, autophagy, endocytosis, catalase activity, stress response to oxidants, lipid metabolism, and reactive oxygen species production. Transcriptome analysis and metabolic profile suggested that AoPex14/17 is involved in multiple cellular processes and the regulation of secondary metabolism. Therefore, our study extends the functions of *PEX* genes, which helps to elucidate the mechanism of organelle development and trap formation in NT fungi and lays the foundation for the development of efficient nematode biocontrol agents.

## INTRODUCTION

Peroxisomes are microbodies, which are organelles enclosed by a single membrane (1). Despite the morphological structure of peroxisomes being simple, their functional diversity is unprecedented. Peroxisomes contain at least 50 kinds of enzymes, such as catalase, peroxidase, and oxidase. As a result, peroxisomes play a role in a number of metabolic processes, such as fatty acid β-oxidation, redox homeostasis, and synthesis of ether glycerolipid plasmalogens (2). Interestingly, peroxisomes have different names in different species due to the differences in their function. For example, in plants, peroxisomes are named glyoxysomes because they contain glyoxylate cycle enzymes (3). In trypanosomes, peroxisomes are called glycosomes because they are involved in the process of glycolysis (4). Furthermore, Woronin bodies (WBs), a specific kind of peroxisome found in filamentous fungi, maintain cellular integrity by shutting the septal pores in response to wounding (5). Notably, various mechanisms (ribosome read-through, differential splicing, and low-efficiency peroxisomal targeting signals) have been demonstrated to achieve partial peroxisomal targeting of glycolytic enzymes in fungi, which contributes to further understanding of the peroxisomal metabolic processes (6). Moreover, proteomic analysis has previously substantially increased our understanding of peroxisomes in some model species, such as plants, yeasts, and mammals (7). However, the distribution and functions of peroxisomes in most filamentous fungi are still poorly understood.

Peroxins are proteins involved in peroxisome biogenesis, and the genes that encode them are known as *PEX* genes (8). Recent advances have identified more than 30 *PEX* genes as crucial factors in peroxisome biogenesis in a variety of species, from yeast to humans (2). Peroxisomal matrix proteins (PMPs) are synthesized on free polyribosomes in the cytosol and posttranslationally imported into organelles (9). PMPs are delivered to peroxisomes via the following two peroxisomal targeting signals (PTSs): type1 (PTS1) and type2 (PTS2). Specific PTS receptors, Pex5 or Pex7, recognize matrix proteins containing PTS1 or PTS2 signals in the cytosol (10). The N terminus of Pex5 can mediate the cargo of the PTS1 sequence through interaction with the docking complex (Pex13, Pex14, and Pex17) (11–13). In addition, Pex18 and Pex21 interact with Pex7 and are key components for targeting the peroxisome in Saccharomyces cerevisiae (14), Pex20 in filamentous fungi (15), and the splice variant Pex5L in plants and mammals (16). Interestingly, the docking complex can also be composed of Pex13, Pex14, and Pex14/17, instead of Pex17 in filamentous fungi (17). Pex14/17, also known as Pex33, distributes in the peroxisomal membrane and interacts with Pex5 and Pex14 (18). The amino acid sequence of the N terminus of Pex14/17 is similar to that of Pex14, whereas its C terminus is similar to Pex17 (19). After docking, the receptor-cargo complex is transferred to the peroxisome matrix, and finally the target protein is released and recycled by the receptor for reuse (20). Recent studies have identified Pex14/17 as peroxin found only in filamentous fungi (21). In Penicillium chrysogenum, the loss of *PEX14/17* affects conidia formation, the penicillin biosynthesis process, and the matrix protein import (19). In Podospora anserina, docked peroxins Pex14 and Pex14/17 are involved in peroxisomal import in different forms (22). In the rice blast fungus Magnaporthe oryzae, the distribution of MoPex14/17 on the peroxisomal membrane is critical for the peroxisomal targeting of PTS1-containing proteins (21). At present, the roles of Pex14/17 in filamentous fungi remain largely unknown.

Nematode-trapping (NT) fungi are a group of filamentous species that use specialized trapping devices (traps), such as constricting rings, adhesive networks, and adhesive knobs to capture, kill, and consume nematodes (23, 24). Therefore, NT fungi can act as natural antagonists of nematodes (21). Arthrobotrys oligospora is the first NT fungus whose genome has been sequenced; it can capture nematodes by producing adhesive networks (25). Previous studies revealed that the cells of the mature trap are generally crowded with “special” microbodies, called electron-dense (ED) bodies, that were not found in normal vegetative hyphae (26). Subsequently, cytochemical staining showed that EDs contained catalase and d-amino acid oxidase, thus EDs were considered as peroxisomal in nature (26). Recently, increasing numbers of studies have suggested that heterotrimeric G-proteins (G-protein) and related signals play an indispensable role in asexual development and lifestyle transition (25), and several autophagy-related genes are also involved in the trap formation of *A. oligospora*, such as *ATG5* (27), and *ATG11* (28). Recently, two *PEX* genes, *PEX1* and *PEX6*, encoding an AAA-type ATPase, were identified in *A. oligospora*, and the absence of traps, conidia, and peroxisomes was caused by the loss of *PEX1* and *PEX6* (29). Thus, peroxisomes are functionally related to mycelium development and trap formation, but the functions of most *PEX* genes in NT fungi remain unclear.

In the current study, we identified a filamentous fungus-specific peroxin Pex14/17 (AoPex14/17) in *A. oligospora* via gene disruption and phenotypic comparison, and the influence of AoPex14/17 on global changes in gene transcription levels was analyzed by RNA sequencing. Meanwhile, the regulation of AoPex14/17 in the secondary metabolism was determined by the metabolome. Our analysis shows that AoPex14/17 is required for trap formation and regulates a variety of phenotypic features, including mycelial development, conidiation, fatty acid utilization, and stress response.

## RESULTS

### Identification of *AoPEX14/17* in *A. oligospora*.

The compute pI/MW tool of the ExPASy server (http://web.expasy.org) predicted that the *AoPEX14/17* encodes a polypeptide of 456 amino acid residues, with a predicted molecular mass of 49.27 kDa and an isoelectric point of 4.78, and AoPex14/17 contained one conserved domain of Pex14_N (IPR006785). AoPex14/17 showed a high similarity (71.83 to 95.14%) to the orthologs of four other NT fungal species, including Arthrobotrys flagrans, Dactylellina haptotyla, Drechslerella stenobrocha, and Drechslerella brochopaga, and it has a moderate similarity to the orthologs of Aspergillus nidulans (44.44%) and other filamentous fungi (29.52 to 27.70%), such as Metarhizium robertsii, Fusarium graminearum, and Aspergillus fumigatus. As expected, Pex14/17 orthologs from NT fungi clustered into an independent branch (see Fig. S1A in the supplemental material).

10.1128/msphere.00012-23.1 FIG S1 Neighbor-joining phylogenetic tree based on the amino acid sequence of Pex14/17 homologous proteins from different fungi and *AoPEX14/17* knockout and verification in *A. oligospora*. (A) Phylogenetic tree. GenBank accession numbers are provided in brackets. Numbers below nodes indicate the bootstrap value. The bar marker indicates the genetic distance, which is proportional to the number. (B) Diagrammatic sketch of homologous recombination. WT, wild-type strain; MT, mutant. (C) The homologous recombinant *AoPEX14/17* deletion plasmid was transformed into yeast. (D) *AoPEX14/17* deletion transformants (Δ*Aopex14/17*) were confirmed by PCR method. 2K, DNA marker. (E) Southern blotting analysis of wild-type and transformants. Download FIG S1, TIF file, 2.1 MB. Copyright © 2023 Liu et al. 2023 Liu et al. https://creativecommons.org/licenses/by/4.0/ This content is distributed under the terms of the Creative Commons Attribution 4.0 International license.

### Disruption of *AoPEX14/17*.

The targeted gene deletion mutants were generated by the homologous recombination method (Fig. S1B). Approximately 60 putative deletion mutants of *AoPEX14/17* were selected on potato-dextrose-agar-sucrose (PDAS) regeneration medium containing hygromycin and identified by genomic PCR and Southern blotting analyses (Fig. S1C to E). Three of the confirmed mutants, Δ*Aopex14/17*-2, Δ*Aopex14/17*-3, and Δ*Aopex14/17*-7, were selected for phenotypic analysis.

### Transcriptomic insights into the regulatory role of *AoPEX14/17*.

The wild-type (WT) and Δ*Aopex14/17* strains were inoculated in PD broth for 5 and 7 days, and then we collected them and performed comparative RNA-seq analysis. A total of about 42.58 to 58.27 million reads were obtained per sample (see Table S1 in the supplemental material). The quality control data show that the base error rates were both less than 0.03%, while the Q20 was greater than 97.72%, the Q30 was greater than 93.62%, and the GC content was greater than 47.57% (Table S1). Principal-component analysis (PCA) suggested the gene expression patterns of WT and mutant strains were distributed in various quadrants at different time points with high similarity, indicating consistency between biological replicates (see Fig. S2A in the supplemental material). The accuracy of the transcriptome data was verified by reverse transcription-quantitative PCR (RT-qPCR). Twenty genes associated with the mitogen-activated protein kinase (MAPK) signaling pathway, glycolysis/gluconeogenesis, and ubiquitin-mediated proteolysis were selected for validation, and the results show that the RT-qPCR results were consistent with transcriptome clustering results (Fig. S2B to D).

10.1128/msphere.00012-23.2 FIG S2 Principal component analysis (PCA) and reverse transcription-qualitative PCR (RT-qPCR) was used to verify the transcriptome data between the WT and Δ*Aopex14/17* mutant strains at 5 days and 7 days. (A) Principal component analysis. Different shapes and colors represent the WT and Δ*Aopex14/17* mutant strains at 5 days and 7 days. (B to D) Comparison of transcription analysis of genes involved in MAPK signaling pathway (B), glycolysis/gluconeogenesis (C), and ubiquitin-mediated proteolysis (D). Download FIG S2, TIF file, 1.8 MB. Copyright © 2023 Liu et al. 2023 Liu et al. https://creativecommons.org/licenses/by/4.0/ This content is distributed under the terms of the Creative Commons Attribution 4.0 International license.

10.1128/msphere.00012-23.8 TABLE S1 Statistics of reads and mapping rate for the wild-type (WT) strain (A) and Δ*Aopex14/17* (H) mutant strains at different time points and statistics of reads, phred-like quality scores, and GC content for the wild-type (WT) strain (A) and Δ*Aopex14/17* (H) mutant at different time points. Download Table S1, DOCX file, 0.02 MB. Copyright © 2023 Liu et al. 2023 Liu et al. https://creativecommons.org/licenses/by/4.0/ This content is distributed under the terms of the Creative Commons Attribution 4.0 International license.

**FIG 1:**
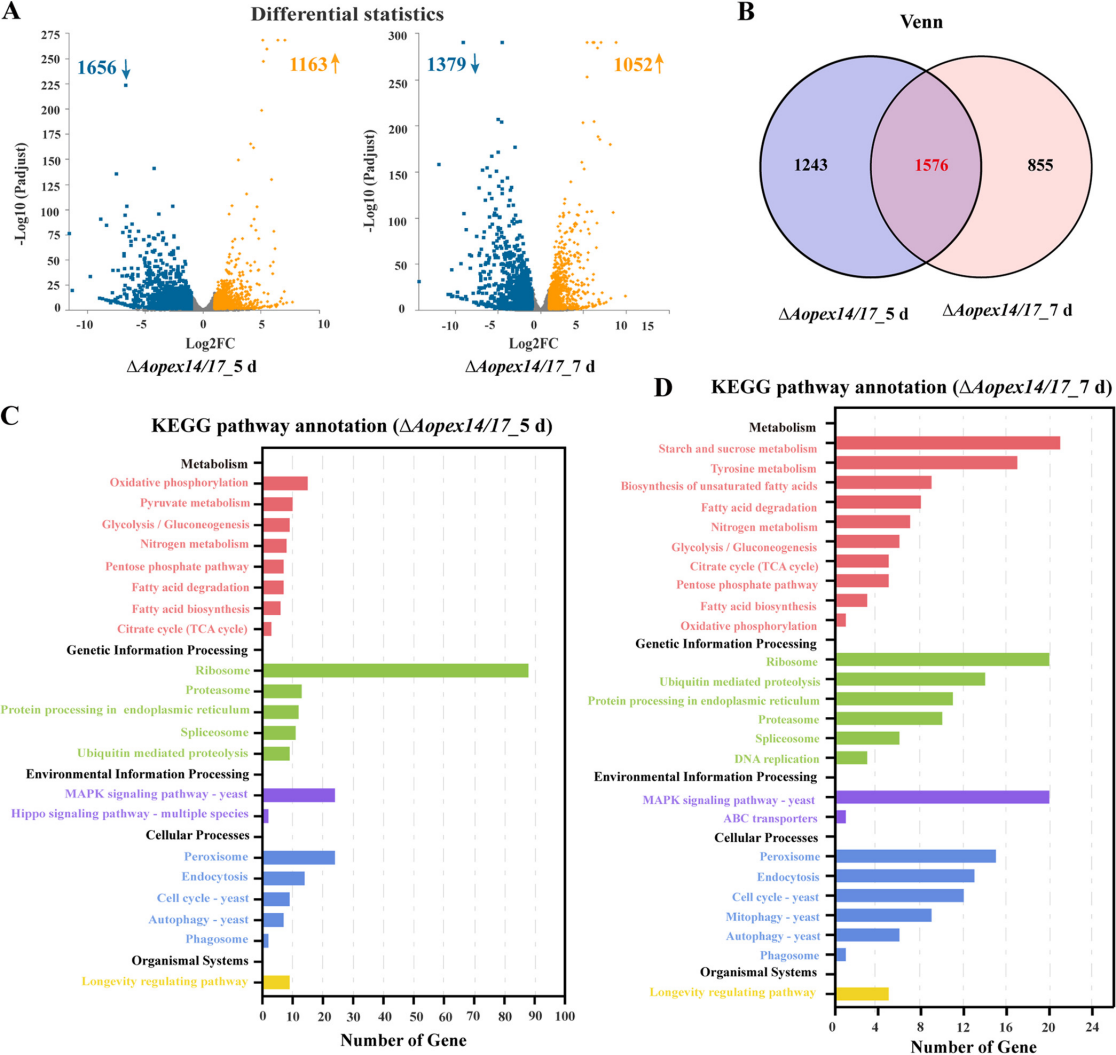
Transcription analysis of wild-type (WT) and Δ*Aopex14/17* mutant strains. (A) Differentially expressed genes (DEGs) on days 5 and 7. Yellow, upregulated DEGs; blue, downregulated DEGs. (B) Venn diagram analysis of DEGs in Δ*Aopex14/17* mutant versus WT strain at 5 and 7 days. (C) KEGG analysis of Δ*Aopex14/17* mutant versus WT strain at 5 days. (D) KEGG analysis of Δ*Aopex14/17* mutant versus WT strain at 7 days. The *x* axis represents the number of genes, and the *y* axis represents the KEGG pathway.

Compared with the WT strain, 2,819 and 2,431 differentially expressed genes (DEGs) were enriched in the Δ*Aopex14/17* strain at 5 and 7 days, respectively. The numbers of upregulated DEGs were 1,163 and 1,052, and those downregulated DEGs were 1,656 and 1,379, respectively (Fig. 1A); Venn analysis displayed that 1,576 DEGs were shared at 5 and 7 days in the Δ*Aopex14/17* strains (Fig. 1B). Kyoto Encyclopedia of Genes and Genomes (KEGG) analysis showed that the DEGs involved in the metabolic pathways were divided into the following five categories: metabolism, genetic information processing, environmental information processing, cellular processes, and organismal systems. Among them, the highest proportion of pathways belonged to the metabolism category. The enrichment results were highly similar between day 5 and day 7 (Fig. 1C and D), with regard to oxidative phosphorylation, glycolysis/gluconeogenesis, nitrogen metabolism, pentose phosphate pathway, fatty acid degradation, fatty acid biosynthesis, citrate cycle (tricarboxylic acid [TCA] cycle), ribosome, proteasome, protein processing in the endoplasmic reticulum, spliceosome, ubiquitin-mediated proteolysis, the MAPK signaling pathway, peroxisome, endocytosis, cell cycle, autophagy, phagosome, and the longevity-regulating pathway. The difference was that pyruvate metabolism in the metabolism category and hippo signaling pathway—multiple species in the environmental information processing category were enriched at 5 days, while starch and sucrose metabolism, tyrosine metabolism and biosynthesis of unsaturated fatty acids, DNA replication in the genetic information processing category, and ABC transporters and mitophagy in the cellular processes category were enriched at 7 days (Fig. 1C and D).

GO enrichment analysis of the DEGs was performed. On the 5th day, the most enriched GO term was biological process (BP), and the top three items of BP were cellular metabolic process (294 unigenes), biosynthetic process (175 unigenes), and oxidation-reduction process (99 unigenes); in the cellular component (CC) category, the top three items were intrinsic component of membrane (384 unigenes), non-membrane-bounded organelle (84 unigenes), and ribosome (72 unigenes); in the molecular function (MF) category, the top items were oxidoreductase activity (101 unigenes), transporter activity (61 unigenes), and RNA binding (41 unigenes) (see Fig. S3A and B in the supplemental material). On the 7th day, in the BP category, the most intrinsic items were metabolic process (303 unigenes), oxidation-reduction process (85 unigenes), and transmembrane transport (65 unigenes); the DEGs enriched in the CC category on day 7 were similar to the membrane intrinsic component (293 unigenes) on day 5; in the MF category, the top three items were catalytic activity (299 unigenes), oxidoreductase activity (87 unigenes), and transporter activity (51 unigenes) (Fig. S3C and D).

10.1128/msphere.00012-23.3 FIG S3 Gene Ontology (GO) enrichment of differentially expressed genes (DEGs) between the WT and Δ*Aopex14/17* mutant strains at 5 days and 7 days. (A, B) GO enrichment analysis of upregulation DEGs (A) and downregulation DEGs (B) in the Δ*Aopex14/17* mutant versus WT strain at 5 days. (C, D) GO enrichment analysis of upregulation DEGs (C) and downregulation DEGs (D) in the Δ*Aopex14/17* mutant versus WT strain at 7 days. Download FIG S3, TIF file, 1.8 MB. Copyright © 2023 Liu et al. 2023 Liu et al. https://creativecommons.org/licenses/by/4.0/ This content is distributed under the terms of the Creative Commons Attribution 4.0 International license.

### *AoPEX14/17* regulates vegetative growth and hyphal morphogenesis.

**FIG 2:**
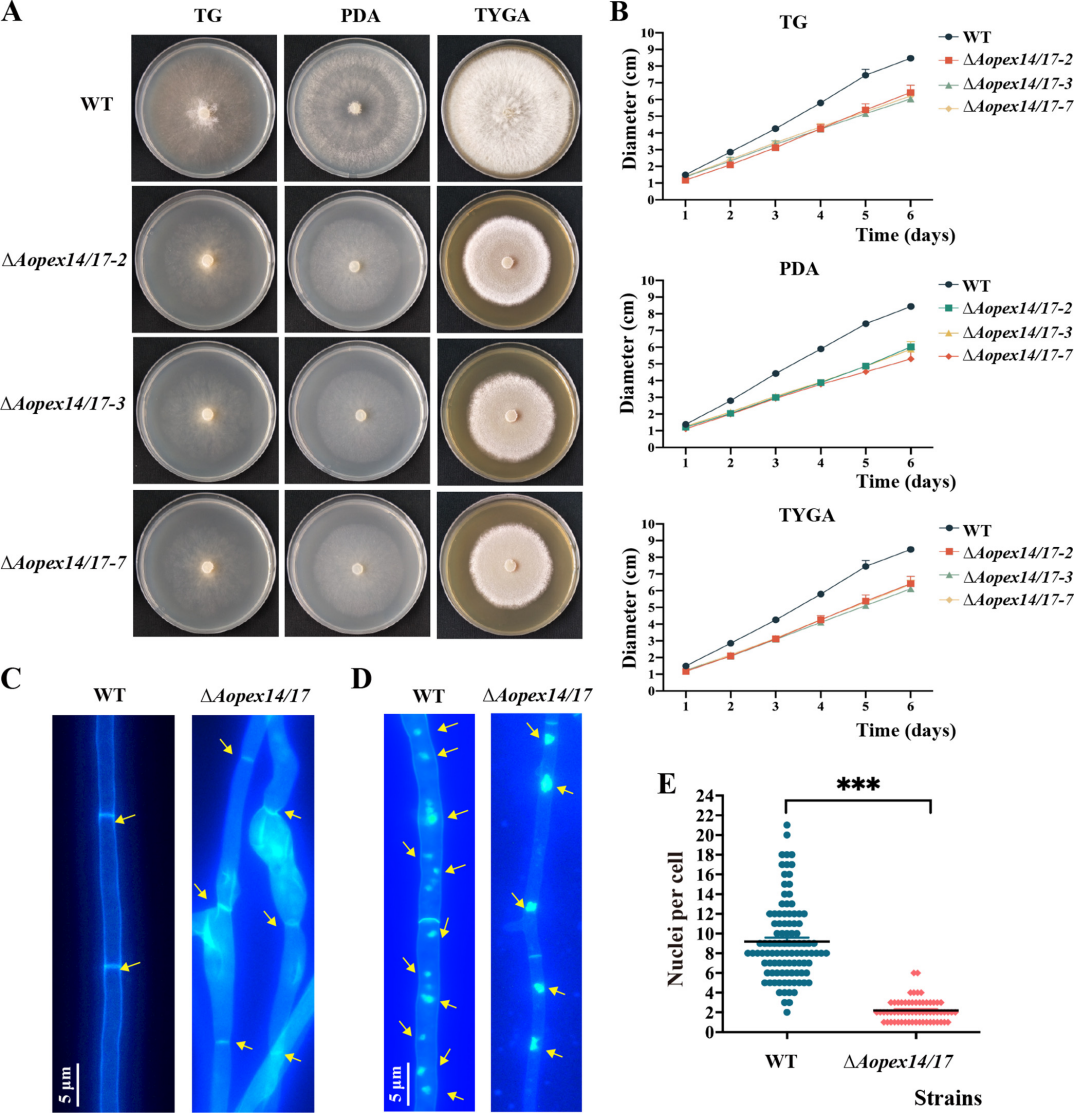
The role of Δ*Aopex14/17* in hyphal growth and morphogenesis. (A) Colony morphology of the WT and Δ*Aopex14/17* strains grown on TG, PDA, and TYGA, respectively. (B) Comparison of colony diameter of the WT and Δ*Aopex14/17* strains. (C) Hyphal septa of the WT and Δ*Aopex14/17* strains were stained with 20 μg/mL calcofluor white (CFW), and the fungal strains were grown for 7 days on CMY medium. Yellow arrows, hyphal septa. Scale bar, 5 μm. (D) The hyphae of the WT and Δ*Aopex14/17* strains were stained with CFW and DAPI, and the fungal strains were grown for 7 days on CMY medium. Yellow arrows, cell nucleus. Scale bar, 5 μm. (E) Comparison of the number of mycelial nuclei between the WT and Δ*Aopex14/17* strains. One hundred hyphal cells were randomly selected for counting cell nuclei. The asterisk indicates a significant difference between the mutant and the WT strains (***, *P* < 0.001).

To characterize the effect of *Aopex14/17* on the mycelium growth and differentiation in *A. oligospora*, we inoculated WT and Δ*Aopex14/17* mutant strains on tryptone-glucose (TG), potato dextrose agar (PDA), and tryptone yeast-extract glucose agar (TYGA) media, respectively, and compared their hyphal growth and colony morphology at 6 days postinoculation. Compared with that of the WT strain, the Δ*Aopex14/17* mutant showed a significant decreased radial growth on all three media (Fig. 2A and B). With regard to the defects in radial growth, we visualized the mycelia septa and morphology using calcofluor white (CFW) staining. The results indicate that the mycelia septum in the Δ*Aopex14/17* mutant was not significantly different from that observed in the WT strain, and partial hyphal cells of the mutant became enlarged (Fig. 2C). Furthermore, the hyphal cells of the WT strain contained 8 to 10 nuclei, whereas the mutants contained 2 to 3 nuclei (Fig. 2D and E).

### *AoPEX14/17* is required for conidiation, trap formation, and pathogenicity.

**FIG 3:**
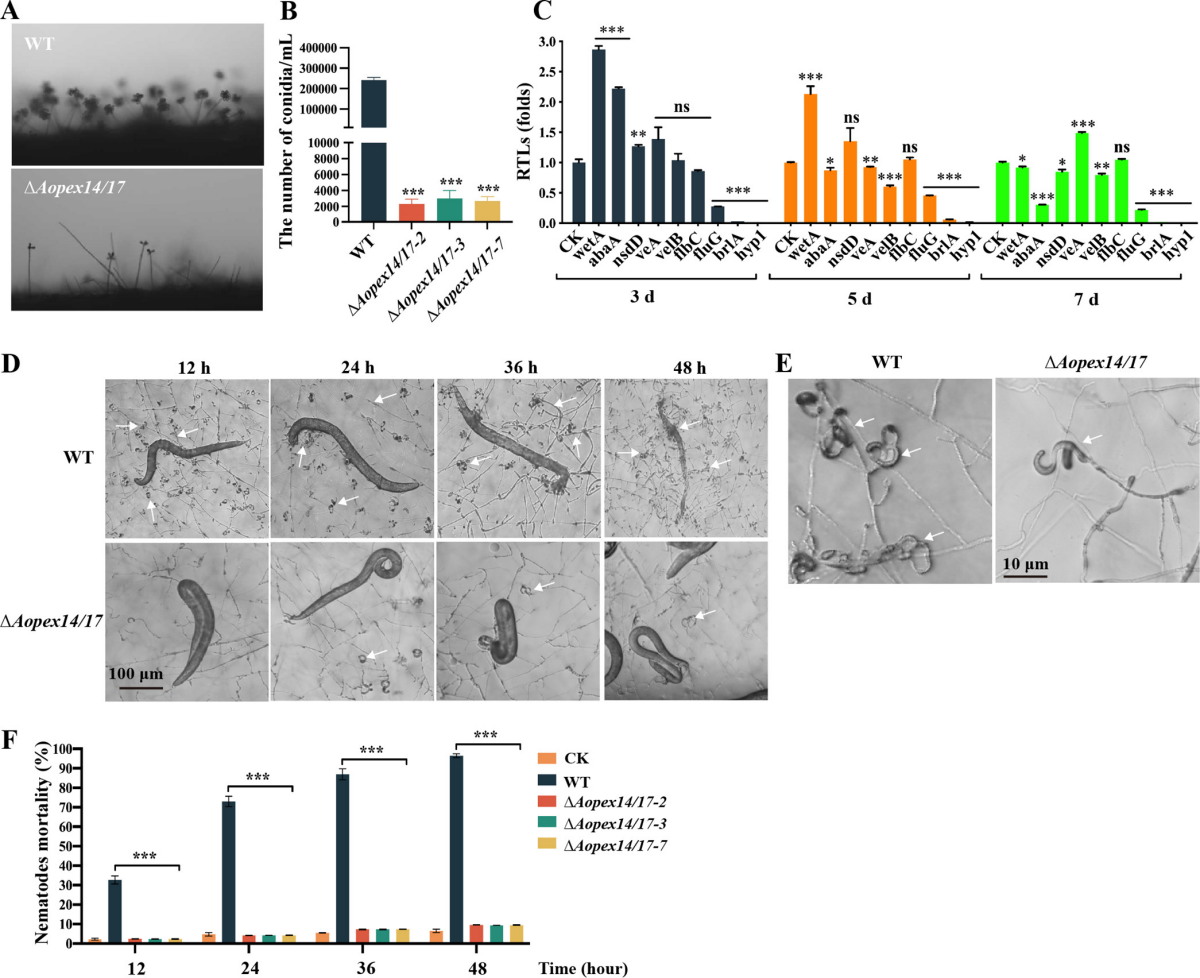
Comparison of conidiation and trap formation between the WT and Δ*Aopex14/17* mutant strains. (A) Observation of conidiophores in the WT and mutant strains. (B) Comparison of conidia yields in the WT and mutant strains. (C) The relative transcription levels (RTLs) of the sporulation-related genes in the WT and Δ*Aopex14/17* mutant strains. (D) Trap formation and nematode predation at different time points. White arrows, traps. Scale bar, 100 μm. (E) Traps produced by the WT and Δ*Aopex14/17* mutant strains at 48 h postinduction. Scale bar, 10 μm. (F) The percentage of nematode mortality in the WT and mutant lines at different time points. CK is the negative control using the WA plate to assess the viability of the C. elegans in the absence of fungal strains (*, *P *< 0.05; **, *P *< 0.01; ***, *P *< 0.001).

The Δ*Aopex14/17* mutant produced approximately 2.0 × 10^3^ to 4.0 × 10^3^ conidia/mL, which is only 1% of that produced by the WT strain (Fig. 3A and B). The RT-qPCR results demonstrate that the expression levels of several genes involved in conidiation from filamentous fungi, including *wetA*, *abaA*, *velB*, *fluG*, *brlA*, and *hyp1*, were significantly downregulated in the Δ*Aopex14/17* mutant (Fig. 3C). Moreover, we assessed the ability of WT and Δ*Aopex14/17* strains to produce traps by inducing with nematodes (Caenorhabditis elegans) to water agar (WA) plates. Our results demonstrate that the Δ*Aopex14/17* mutant was unable to produce mature traps (Fig. 3D). In the Δ*Aopex14/17* mutant, half rings or one ring appeared at 12 h, one and one-half ring appeared at 24 h, two rings appeared at 36 h, and three rings appeared at 48 h, but no mature traps were formed until 5 days (Fig. 3D and E). Additionally, the nematode mortality of the WT strain was as high as 96% at 48 h, whereas the nematode mortality of the Δ*Aopex14/17* strain was less than 10% at each time point (Fig. 3F). Thus, deletion of the *AoPEX14/17* gene significantly reduced the ability of the strain to infect nematodes.

### *AoPEX14/17* contributes to stress tolerance.

**FIG 4:**
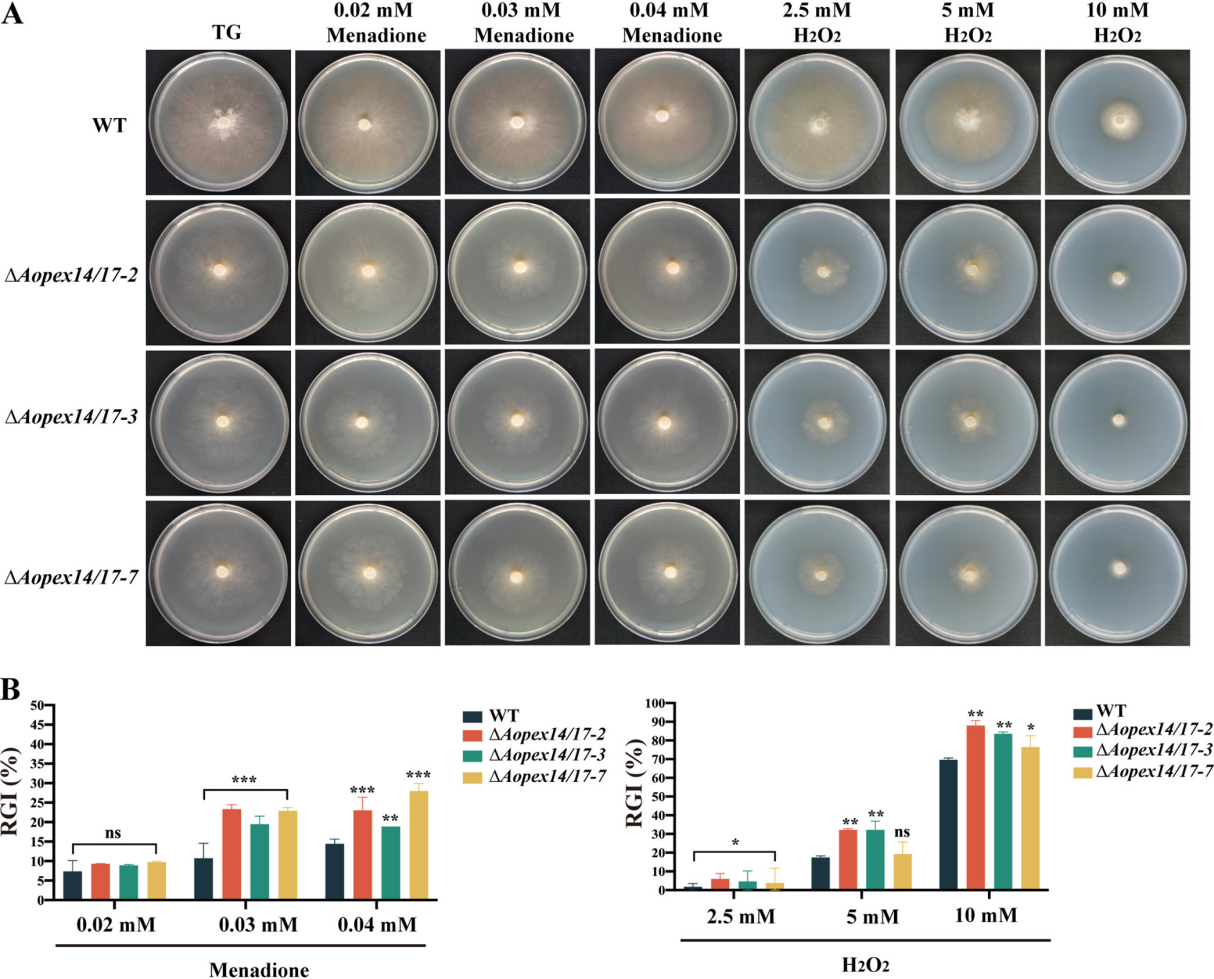
Comparison of stress tolerance to oxidative agents between the WT and Δ*Aopex14/17* mutant strains. (A) Colony morphologies of the WT and mutant strains incubated on TG medium supplemented with 0.02 to 0.04 mM menadione and 2.5 to 10 mM H_2_O_2_ for 6 days. (B) Relative growth inhibition (RGI) values of the WT and mutant strains incubated on TG medium supplemented with 0.02 to 0.04 mM menadione and 2.5 to 10 mM H_2_O_2_ for 6 days. The asterisk indicates a significant difference between the mutant and the WT strains (*, *P *< 0.05; **, *P *< 0.01; ***, *P *< 0.001).

To determine the function of *Aopex14/17* in stress response, we treated strains to compounds that stimulate oxidative (menadione and H_2_O_2_), cell wall (Congo red [CR] and sodium dodecyl sulfate [SDS]), and osmotic stress (NaCl and sorbitol). The Δ*Aopex14/17* strain was sensitive to high concentrations of menadione and H_2_O_2_, and the relative growth inhibition (RGI) values were significantly higher than those of the WT strain (Fig. 4A and B). In the transcriptome analysis, eight genes involved in oxidative stress response were significantly decreased on day 5 and day 7, including *cat* (AOL_s00173g374 and AOL_s00188g243), *cat-2* (AOL_s00006g411 and AOL_s00076g488), *nox-1*, *nox-2*, *sod*, *sodB*, and *sod-2* (Table 1).

**TABLE 1:** Transcriptional response to *Aopex14/17* deletion by the genes involved in oxidation stress response and cell wall biosynthesis in comparative transcriptome analysis

Function/locus	Function annotation	Expressional level^*a*^			
		TPM (5 days)		TPM (7 days)	
		WT	Δ*Aopex14/17* strain	WT	Δ*Aopex14/17* strain
Genes involved in oxidation stress response					
AOL_s00173g374	*cat*, catalase	508.06	144.38	922.10	374.65
AOL_s00188g243	*cat*, catalase	0	0.08	0	0.013
AOL_s00006g411	*cat2*, catalase	383.91	60.26	14.17	15.47
AOL_s00076g488	*cat2*, catalase	14.81	7.13	105.93	61.40
AOL_s00193g69	*nox-1*, NADPH oxidase	166.42	52.987	485.51	43.85
AOL_s00007g557	*nox-2*, NADPH oxidase	70.64	37.45	51.46	22.41
AOL_s00054g538	*noxR*, NADPH oxidase regulator	2.68	6.56	3.81	11.30
AOL_s00007g292	*sod*, superoxide dismutase	88.75	46.85	165.99	121.24
AOL_s00054g687	*sodB*, superoxide dismutase	608.14	180.58	794.69	281.04
AOL_s00170g93	*sod-2*, superoxide dismutase	8.62	5.65	5.18	2.27
Genes involved in cell wall biosynthesis					
AOL_s00078g76	*chs-3*, chitin synthases	93.29	40.57	126.43	61.53
AOL_s00112g89	*hex*, hexokinase	119.19	244.53	178.62	255.77
AOL_s00075g119	*chi*, chitin synthase	66.42	4.86	28.14	8.70
AOL_s00097g268	*trs*, trehalose synthase	246.41	191.29	156.55	65.34
AOL_s00083g375	*glu*, beta-glucosidase	8.20	27.44	9.65	37.66
AOL_s00054g491	*gls*, 1,3-beta-glucan synthase	184.01	66.87	245.77	71.90

a TPM, transcripts per kilobase million; WT, wild-type strain; Δ*Aopex14/17*, *AoPEX14/17* deletion mutant; 5 days and 7 days, vegetative growth stage. Locus numbers and function were annotated according to the *A. oligospora* genome assembly (https://www.ncbi.nlm.nih.gov/).

Moreover, the RGI value of the Δ*Aopex14/17* mutant was higher than that of the WT strain under stress of SDS (0.01-0.03%), and the Δ*Aopex14/17* mutant showed a decreased RGI value compared to the WT strain at 0.1 mg/L CR (see Fig. S4A and B in the supplemental material). Similarly, the Δ*Aopex14/17* mutant showed a higher RGI value than the WT strain at 0.3 M NaCl, whereas no obvious defects in stress responses to sorbitol were observed (Fig. S4C and D). We also examined the expressions of genes involved in cell wall biosynthesis in the transcriptome; except for *hex* and *glu*, the expression levels of other genes were significantly decreased on day 5 and day 7, including *chs-3*, *chs*, *trs*, and *gls* (Table 1).

10.1128/msphere.00012-23.4 FIG S4 Comparison of stress tolerance to cell wall-perturbing agents and osmotic agents between the WT and Δ*Aopex14/17* mutant strains. (A) Colony morphologies of the WT and mutant strains incubated on TG medium supplemented with 0.05 to 0.1 mg/mL Congo red and 0.01 to 0.03% SDS for 6 days. (B) Relative growth inhibition (RGI) values of the WT and mutant strains incubated on TG medium supplemented with 0.05 to 0.1 mg/mL Congo red and 0.01 to 0.03% SDS for 6 days. (C) Colony morphologies of the WT and mutant strains incubated on TG medium supplemented with 0.10 to 0.30 M NaCl and 0.25 to 0.75 M sorbitol for 6 days. (D) Relative growth inhibition (RGI) values of the WT and mutant strains incubated on TG medium supplemented with 0.10 to 0.30 M NaCl and 0.25 to 0.75 M sorbitol for 6 days. The asterisk indicates a significant difference between the mutant and the WT strains (*, *P *< 0.05; **, *P *< 0.01). Download FIG S4, TIF file, 7.6 MB. Copyright © 2023 Liu et al. 2023 Liu et al. https://creativecommons.org/licenses/by/4.0/ This content is distributed under the terms of the Creative Commons Attribution 4.0 International license.

### *AoPEX14/17* regulates intracellular autophagy, ROS accumulation, and endocytosis.

**FIG 5:**
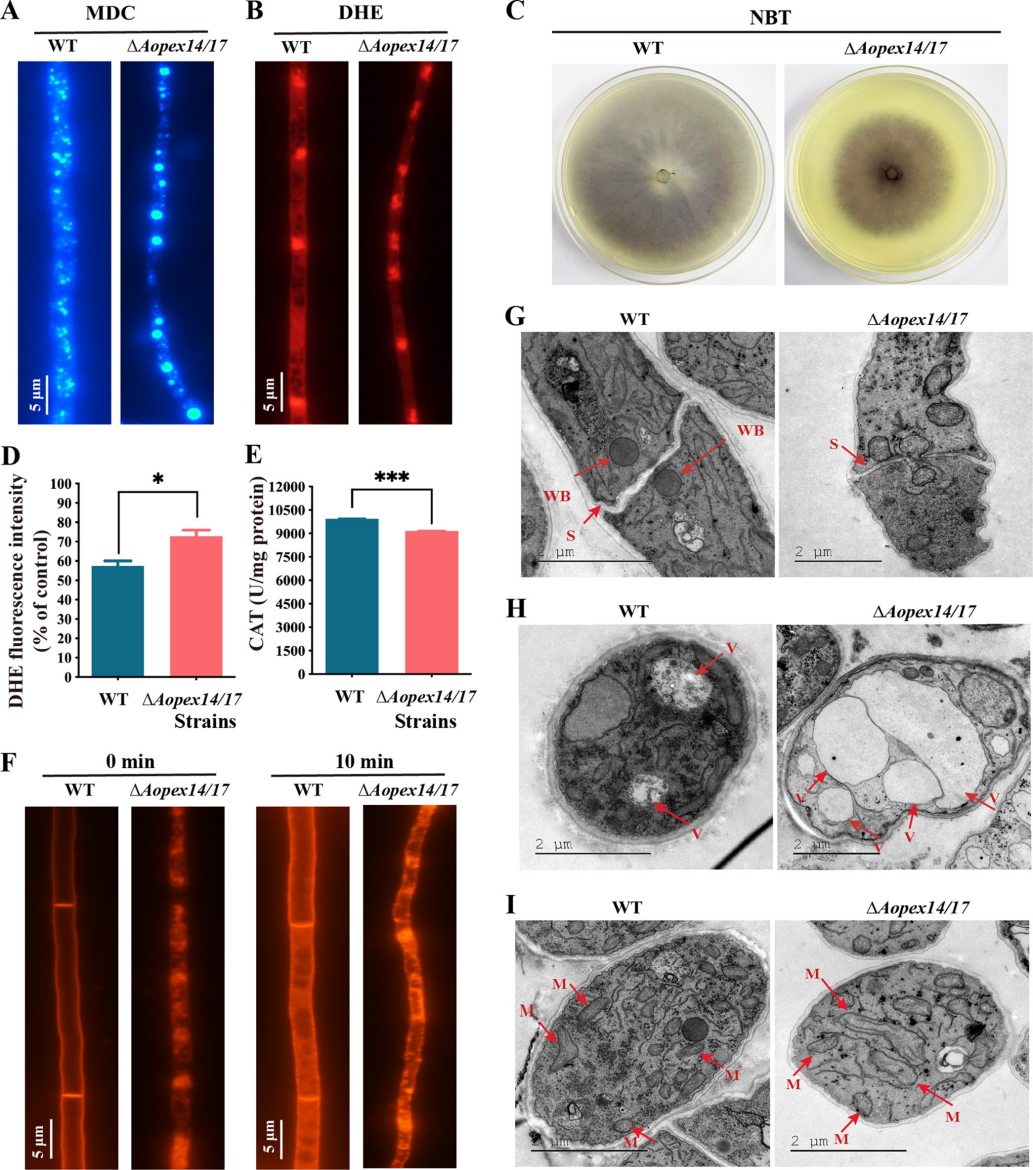
Observation of intracellular autophagosomes, ROS accumulation, endocytosis, Woronin bodies, and vacuoles between the WT and Δ*Aopex14/17* mutant strains. (A) The autophagosomes of the WT and Δ*Aopex14/17* mutant strains were stained with monodansylcadaverine (MDC). Scale bar, 5 μm. (B) Representative images of dihydroethidium (DHE) staining to detect superoxide in the WT and Δ*Aopex14/17* mutant strains. Scale bar, 5 μm. (C) Nitroblue tetrazolium (NBT) staining for ROS production in mycelia of the WT and Δ*Aopex14/17* mutant strains. The dark color of the colony indicates increased ROS production. (D) Analysis of the DHE fluorescence intensity of the same weight of mycelium in the WT and Δ*Aopex14/17* mutant strains via multimode microplate reader. (E) Catalase (CAT) activity assay of the same weight of mycelium in the WT and Δ*Aopex14/17* mutant strains via multimode microplate reader. (F) FM4-64 staining to observe endocytosis in the WT and Δ*Aopex14/17* mutant strains at different times. Scale bar, 5 μm. (G to I) The WT and Δ*Aopex14/17* mutant strains were observed by transmission electron microscopy. WB, Woronin body; S, septum; V, vacuoles; M, mitochondrion. Scale bar, 2 μm.

Monodansylcadaverine (MDC) staining was used to monitor the autophagy process, and autophagosomes in hyphae displayed blue fluorescence after MDC staining (30). Compared with that of WT, the Δ*Aopex14/17* strain showed an increased accumulation of autophagosomes (Fig. 5A). Accordingly, 10 and 17 genes related to autophagy were differentially expressed at days 5 and 7, respectively, and three genes encoding cell division control protein Cdc48 (AOL_s00004g527) and serine protease (AOL_s00170g103 and AOL_s00176g50) were upregulated in the Δ*Aopex14/17* strain at both day 5 and day 7 by transcriptome analysis (see Fig. S5A in the supplemental material).

10.1128/msphere.00012-23.5 FIG S5 Differentially expressed genes (DEGs) associated with autophagy and peroxisome. Download FIG S5, TIF file, 1.2 MB. Copyright © 2023 Liu et al. 2023 Liu et al. https://creativecommons.org/licenses/by/4.0/ This content is distributed under the terms of the Creative Commons Attribution 4.0 International license.

After staining with dihydroethidium (DHE) and nitrotetrazolium blue chloride (NBT), the hyphae of the Δ*Aopex14/17* strain were more intensely stained by NBT than the WT strain, indicating greater reactive oxygen species (ROS) accumulation in the Δ*Aopex14/17* strain (Fig. 5B and C). Moreover, the fluorescence intensity levels of DHE in the WT and Δ*Aopex14/17* strains were 57.5 and 69.5, respectively (Fig. 5D). Similarly, we determined catalase (CAT) activity in the WT and Δ*Aopex14/17* strains. The results show that the activity of CAT was reduced in the Δ*Aopex14/17* strain (Fig. 5E). This is consistent with our transcriptome analysis of the significantly reduced expression levels of CAT activity-related genes *cat* and *cat-2* (Table 1).

To clarify the role of *AoPEX14/17* in endocytosis, we stained the hyphal cells of WT and Δ*Aopex14/17* strains with FM4-64 and followed the uptake of the dye over time. In the WT strain, the dye first appeared on the plasma membrane and was taken up by the cells after 10 min. In contrast, in the Δ*Aopex14/17* strain, the dye uptake was accelerated and localized to the cytoplasm, which may be mature endosomes or vacuoles (Fig. 5F).

### *AoPEX14/17* is required for the formation of WBs and impairs biogenesis of other organelles.

Transmission electron microscopy (TEM) revealed that WBs were observed near the septa in the hyphae of the WT, whereas they were not observed in Δ*Aopex14/17* hyphae (Fig. 5G), and the hyphal septum of the Δ*Aopex14/17* strain was broken up, consistent with the role of WBs that prevent the multiple organelles and intracellular substance from effluxing (Fig. 5G). We also found that the volume and number of vacuoles were significantly increased in the Δ*Aopex14/17* strain compared with those of the WT strain (Fig. 5H). However, the number of mitochondria and mitochondrial cristae decreased significantly, and the mitochondrial shape became longer, wider, and vacuolated in the Δ*Aopex14/17* hyphae (Fig. 5I).

Moreover, we performed cluster analysis of peroxisome-related genes. The expression levels of five genes associated with peroxisome biosynthesis were upregulated both at day 5 and day 7, including peroxisome membrane proteins (Pex4, AOL_s00078g269), Pmp47 (AOL_s00215g205), MPV17 (AOL_s00215g316), peroxin-2 (Pex2, AOL_s00080g243), peroxisomal targeting receptor (Pex5, AOL_s00043g671), and four genes relate to fatty acid degradation, including enoyl-coenzyme A (enoyl-CoA) hydratase (AOL_s00215g56 and AOL_s00043g730), acetyl-CoA acyltransferase (AOL_s00210g122), and carnitine *O*-acetyltransferase (AOL_s00109g5). In addition, four genes were downregulated at 5 and 7 days, namely, integral membrane protein (AOL_s00006g182), superoxide dismutase (SodB, AOL_s00054g687), and sarcosine oxidase (AOL_s00117g57), and we also clustered a marked decrease in the expression level of *AoPEX14/17*, consistent with which *Aopex14/17* is knocked out (Fig. S5B).

### *AoPEX14/17* deletion led to defects in lipid metabolism.

**FIG 6:**
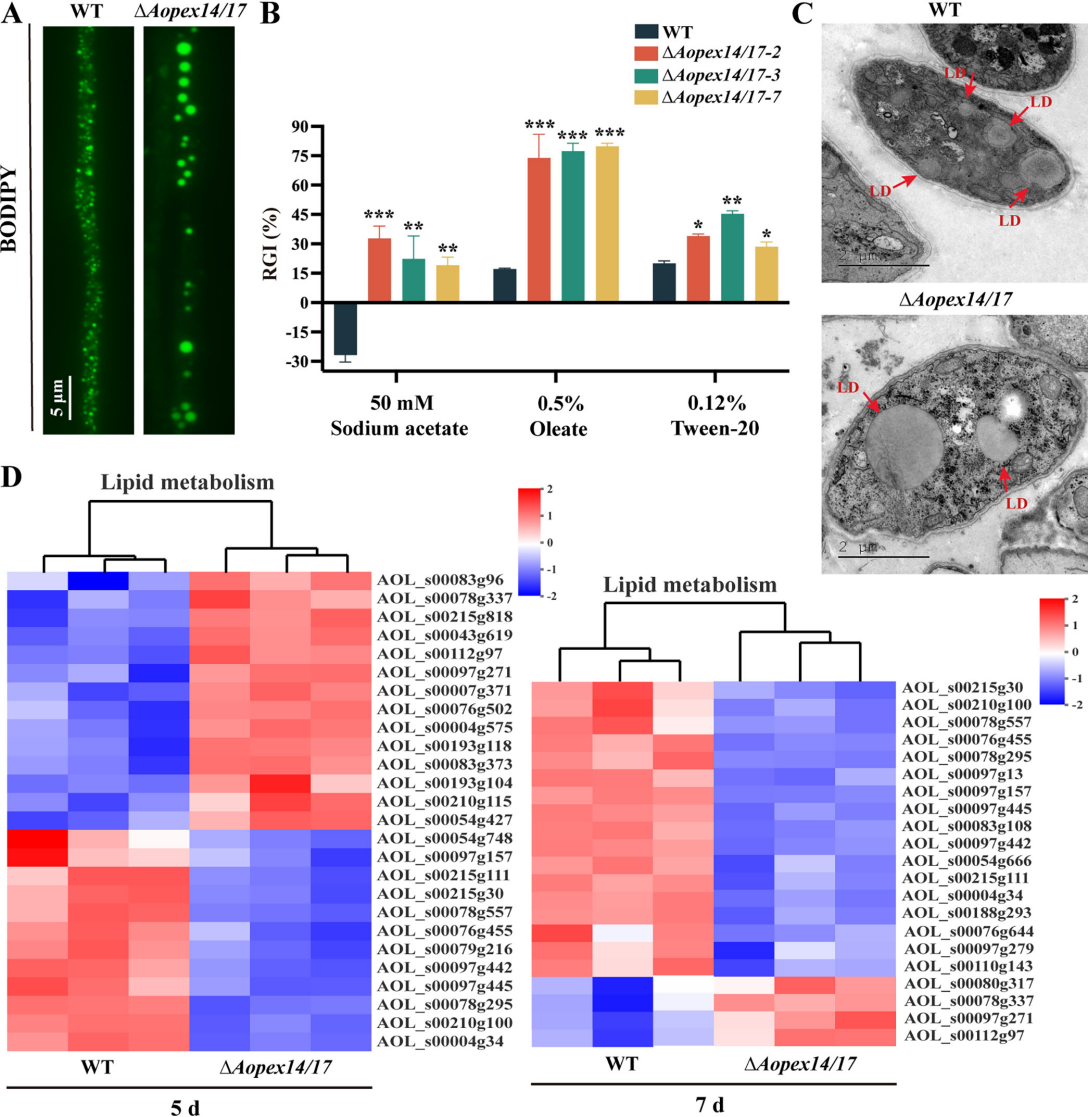
Comparison of lipid metabolism between the WT and Δ*Aopex14/17* mutant strains. (A) The LDs of the WT and Δ*Aopex14/17* mutant strains were stained with BODIPY. Scale bar, 5 μm. (B) Relative growth inhibition (RGI) values of fungal strains under different fatty acids as the only carbon source. (C) The lipid droplets (LDs) of the WT and Δ*Aopex14/17* mutant strains were observed by transmission electron microscopy. Scale bar, 2 μm. (D) Differentially expressed genes (DEGs) associated with lipid metabolism (*, *P *< 0.05; **, *P *< 0.01; ***, *P *< 0.001).

Peroxisome plays a significant role in fatty acid β-oxidation (31). We investigated whether *AoPEX14/17* affects fatty acid level by staining with the neutral lipid-specific fluorescent dye boron dipyrromethene (BODIPY). The number of lipid droplets (LDs) in the Δ*Aopex14/17* strain was reduced, but the volume was increased compared to that of the WT strain (Fig. 6A). To investigate the role of *AoPEX14/17* in fatty acid utilization, the WT and Δ*Aopex14/17* mutant strains were inoculated on MM plates containing short-chain (sodium acetate), medium-chain (Tween 20), or long-chain (oleate) fatty acids as the sole carbon source, respectively (Fig. 6B). After 6 days, the growth rate of the Δ*Aopex14/17* strain was remarkably reduced on all three plates compared to the WT strain. The RGI values of the Δ*Aopex14/17* strain on plates supplemented with all three fatty acids were markedly increased (Fig. 6B). Additionally, we also observed larger LDs in the hyphae of the Δ*Aopex14/17* strain by TEM (Fig. 6C).

Moreover, we conducted a transcriptome clustering analysis of the genes involved in lipid metabolism. The results show that nine downregulated genes were shared at day 5 and day 7, including glycerophosphocholine phosphodiesterase (AOL_s00097g157), ceramide glucosyltransferase (AOL_s00215g111), phospholipase D (PldA) (AOL_s00215g30), 1-alkyl-2-acetylglycerophosphocholine esterase (AOL_s00078g557), alkaline ceramidase (AOL_s00076g455), ethanolamine phosphotransferase (AOL_s00079g216), choline kinase (AOL_s00097g442), auxin efflux carrier superfamily protein (AOL_s00097g445), ethanolamine kinase (AOL_s00078g295), phospholipase A2 (PlaA) (AOL_s00210g100), and hypothetical protein (AOL_s00004g34) (Fig. 6D).

### *AoPEX14/17* involvement in the regulation of the biosynthesis of secondary metabolites.

**FIG 7:**
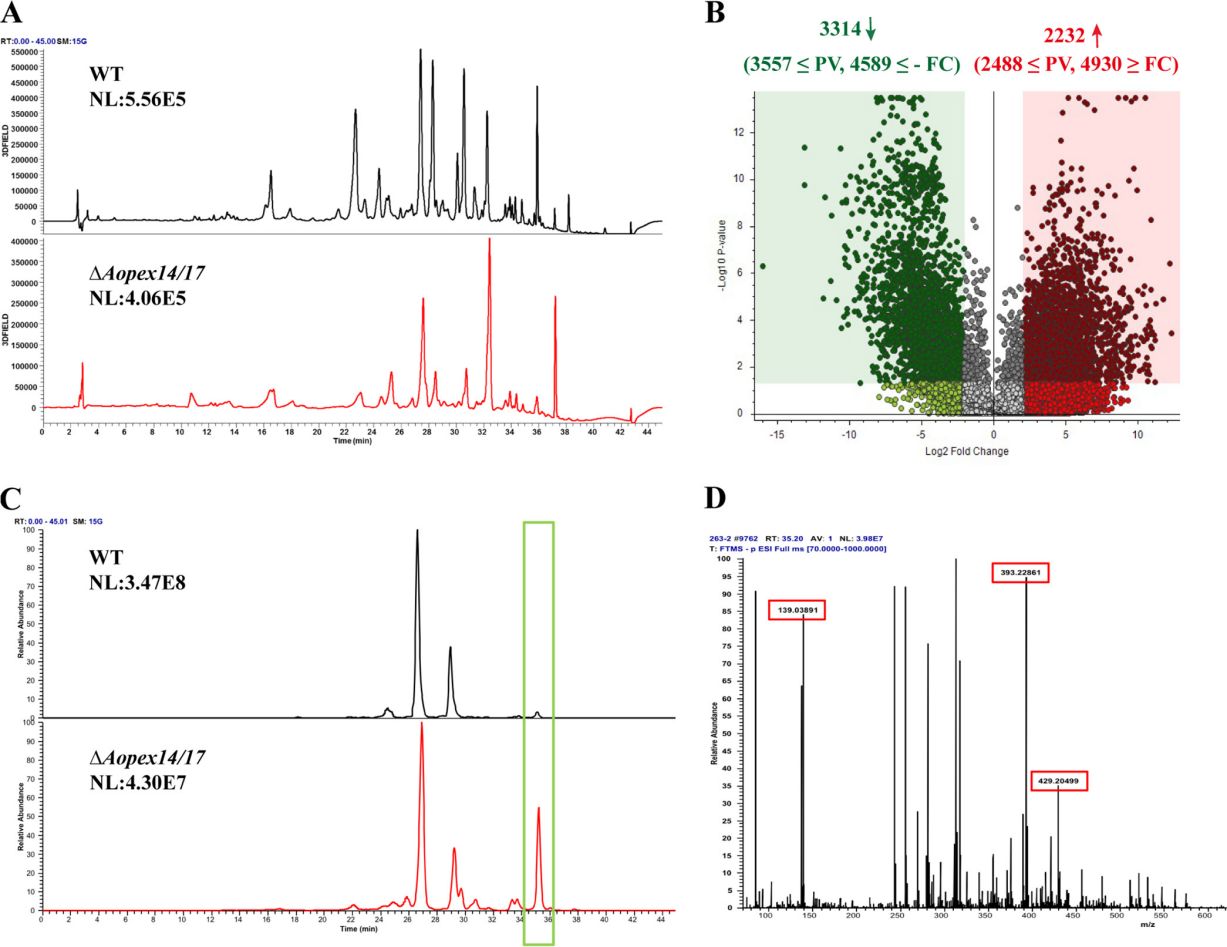
Comparison of metabolic profiling between the WT and Δ*Aopex14/17* mutant strains. (A) Comparison of high-performance liquid chromatography profiles of the WT and Δ*Aopex14/17* mutant strains. (B) Volcano plot of differential metabolites between the WT and Δ*Aopex14/17* mutant strains. (C) LC-MS analysis showing ion chromatogram in the WT and Δ*Aopex14/17* mutant strains. Green frame, the peak of arthrobotrisin. (D) Mass spectrogram of arthrobotrisins in the Δ*Aopex14/17* mutant strain (diagnostic fragments ion at *m/z* 139, 393, and 429). RT = 35.2 min.

High-performance liquid chromatography (HPLC) results indicate that the metabolic abundance produced by the Δ*Aopex14/17* strain was significantly less than that of the WT strain, and the differential peaks were mainly concentrated at retention times (Rt) of 20 to 38 min (Fig. 7A). There were 2,232 upregulated and 3,314 downregulated compounds in the Δ*Aopex14/17* mutant compared with those of the WT (Fig. 7B; see also Fig. S6A and B in the supplemental material), and the top 20 differential compounds are listed in Table S2 in the supplemental material. KEGG analysis of the differential metabolic pathways between WT and Δ*Aopex14/17* strains showed that the upregulated pathways were involved in lipoxygenase, trichothecene biosynthesis, scopolin and esculin biosynthesis, aromatic compounds, etc. (Fig. S6C and Table S2), and the downregulated pathways mainly included aerobic toluene degradation, steroid hormone biosynthesis, rosmarinic acid degradation biosynthesis, and scopolin and esculin biosynthesis (Fig. S6D and Table S2). Ultraperformance liquid chromatography with diode array detection mass spectrometry (UPLC-DAD/MS) spectral comparison showed that the ion peak at Rt 35 min was much higher in Δ*Aopex14/17* strains than in WT (Fig. 7C). MS analysis of this peak revealed a diagnosis of arthrobotrisins, a specific metabolite of *A. oligospora*, at *m/z* 139, 393, and 429 (Fig. 7D).

10.1128/msphere.00012-23.6 FIG S6 Comparison of secondary metabolites between the WT and Δ*Aopex14/17* mutant strains. (A, B) Volcanograms (A) and heatmap (B) of upregulated and downregulated compounds between the WT and Δ*Aopex14/17* mutant strains. (C, D) The metabolic pathways of significantly upregulated (C) and downregulated (D) between the WT and Δ*Aopex14/17* mutant strains determined via KEGG enrichment. Download FIG S6, TIF file, 2.0 MB. Copyright © 2023 Liu et al. 2023 Liu et al. https://creativecommons.org/licenses/by/4.0/ This content is distributed under the terms of the Creative Commons Attribution 4.0 International license.

10.1128/msphere.00012-23.9 TABLE S2 The top 20 compounds with significant changes and the top 10 metabolic pathways that were significantly upregulated and downregulated. Download Table S2, DOCX file, 0.02 MB. Copyright © 2023 Liu et al. 2023 Liu et al. https://creativecommons.org/licenses/by/4.0/ This content is distributed under the terms of the Creative Commons Attribution 4.0 International license.

### AoPex14/17 can interact with AoPex14 but not with AoPex13.

We attempted to probe the interaction between AoPex14/17 and two other docking complex members, AoPex13 and AoPex14, by yeast two-hybrid (Y2H) analysis. Firstly, we chose positive controls including plasmids pGBKT7-53 and pGADT7-T, while negative controls included pGBKT7-Lam and pGADT7-T. Furthermore, the growth of transformants was measured by serial dilution of yeast cells on SD/Trp/Leu, SD/Trp/Leu/His/Ade, and SD/Trp/Leu/His/Ade/-bromo-4-chloro-3-indolyl-β-D-galactopyranoside (X-Gal) media, respectively. Ultimately, the results of Y2H experiments reflect that AoPex14/17 does not interact with AoPex13 but is able to interact with AoPex14 (see Fig. S7A and B in the supplemental material).

10.1128/msphere.00012-23.7 FIG S7 Yeast two-hybrid (Y2H) assays detect the interaction of AoPex14/17 with the other two members of the docking complex, AoPex13 and AoPex14, in *A. oligospora*. (A) Y2H analysis of AoPex14/17 and AoPex13. (B) Y2H analysis of AoPex14/17 and AoPex14. Download FIG S7, TIF file, 6.4 MB. Copyright © 2023 Liu et al. 2023 Liu et al. https://creativecommons.org/licenses/by/4.0/ This content is distributed under the terms of the Creative Commons Attribution 4.0 International license.

## DISCUSSION

The maintenance of the peroxisome depends on the formation of the peroxisomal membrane and the import of both membrane and matrix proteins (18). The matrix proteins that are translocated to the peroxisomal membrane must interact with the docking machinery components, involving multiple peroxins, such as Pex1, Pex2, Pex5, Pex6, Pex7, Pex10, Pex12, Pex13, Pex14, and Pex26 (32, 33). Pex14/17, a filamentous fungus-specific peroxin of the docking machinery of peroxisomes, is required for the import of PMPs via binding Pex5 and Pex14 and may act as ScPex14 and ScPex17 in the docking of the peroxisomal matrix by expressing *PEX14*- and *PEX17*-similar portions of the proteins (19, 22). In this study, we first characterized the presence of homologs of *PEX14/17* in the NT fungus *A. oligospora* and found that it is essential for the regulation of functions of peroxisomes and has a significant impact in a variety of phenotypic features through the disruption of *AoPEX14/17*.

Peroxisome has been linked to the growth, development, and pathogenicity of fungi (29). In Penicillium chrysogenum, the loss of *PEX14/17* leads to a defect in sporulation (19). In M. oryzae, the loss of *PEX14/17* delayed conidial generation and appressorial formation, reduced appressorial turgor accumulation and penetration ability in host plants, and significant decreased fungal virulence; however, *PEX14/17* deletion showed no significant difference with regard to colony morphology and aerial hyphal development and only a slight reduction in radial growth (21). In *M. robertsii*, the conidial germination, conidiation, and fungal virulence were all greatly reduced upon *PEX14/17* depletion, but there were no appreciable changes between colony phenotypes and the colony diameter of the *PEX14/17* mutant strains and control strains on various conditions in the vegetative growth assay (34). In this study, we provide direct evidence that the knockout of *PEX14/17* represses conidial production and germination and impairs trap formation and pathogenicity in *A. oligospora*, which are consistent with previous reports. Comparatively, the loss of *AoPEX14/17* significantly reduces hyphae growth on the three different media. The reduced nuclei involved in the regulation of meiotic recombination (35) and the enrichment of the cell cycle indicated by transcriptome analysis indicate that AoPex14/17 may play a positive role in vegetative growth in *A. oligospora*. In addition, the morphology of the hyphae was changed in the Δ*Aopex14/17* mutants, including inflated mycelial cells. These results indicate that AoPex14/17 is necessary for mycelial growth, conidial production and germination, and the pathogenicity of *A. oligospora* and other filamentous fungi.

In fungi, besides common functions including the β-oxidation of fatty acids and the detoxification of hydrogen peroxide, peroxisomes are crucial for the metabolism of various unique carbon and nitrogen sources, including oleic acid, methanol, primary amines, and uric acid, and play an important role in response to stress (36, 37). Previous evidence has highlighted a decreasing ability for the control of fatty acid utilization, ROS degradation, stress tolerance, and cell wall integrity in fungi without Pex14/17 (21, 34, 38). Our results indicate that the disruption of *AoPEX14/17* also resulted in a significant decrease in fatty acid utilization and tolerance to oxidative stress. In transcriptome analysis, there are nine genes involved in lipid metabolism that were downregulated in the Δ*Aopex14/17* strain versus WT strain, such as glycerophosphocholine phosphodiesterase, phospholipase D, alkaline ceramidase, and ethanolamine kinase; these genes are mainly involved in fatty acid catabolic processes, such as glycerophospholipid metabolic pathways and hydrolases and transferases of various lipids (39, 40). Obviously, the hyphae of Δ*Aopex14/17* mutants exhibited bigger lipid droplets and a much greater accumulation of ROS associated with the lower enzyme activity of CAT. Moreover, Pex14/17 plays a crucial role in peroxisome biogenesis (21). These results reveal that the downregulation of peroxisomal functions caused by a lack of *AoPEX14/17* was triggered by a defect of peroxisome biogenesis in *A. oligospora*. However, no obvious inhibition was observed in Δ*Aopex14/17* mutants under chemical stressors of CR, NaCl, and sorbitol, indicating that AoPex14/17 may play a dispensable role in cell wall integrity and hyperosmotic stresses in *A. oligospora*.

Intriguingly, we observed that the terms of autophagy, mitophagy, and ubiquitin-mediated proteolysis were significantly enriched by transcriptome analysis, and subsequently, the remarkable increase in the level of autophagosome was confirmed by fluorescent staining in the Δ*Aopex14/17* mutants. Autophagy plays a vital role in the maintenance of peroxisome mass, and autophagy further regulates peroxisomes and contributes to the production of ROS (41). Peroxisome abundance can be regulated by *PEX* genes. The upregulation of autophagy level may be caused by the shortage of integrated peroxisome in Δ*Aopex14/17* mutants. Whether the regulation of *PEX14/17* in autophagy and endocytosis inhibits the peroxisome function or alters matrix protein transport remains unknown. In addition, Δ*Aopex14/17* mutants generally increase the endocytosis process but lack WBs, which are peroxisome-related organelles necessary for sealing the septal pore in the event of hyphal damage (42, 43). Consistently, our transcriptome data suggest that Pex14/17 affects multiple metabolic pathways involved in carbohydrate metabolism, the MAPK signaling pathway, and mitochondrial function. TEM analysis revealed that the number of mitochondria and mitochondrial cristae decreased significantly in the Δ*Aopex14/17* mutants. Our findings support a model in which cellular energy metabolism relies heavily on the peroxisome, and the structure and function of peroxisomes and mitochondria are closely related in *A. oligospora*. The metabolome data also show that the abundance of secondary metabolites was significantly reduced upon *PEX14/17* deletion in *A. oligospora*. Fungal secondary metabolites are synthesized by complex biosynthetic pathways catalyzed by enzymes located in different subcellular compartments, including the peroxisome (44). In Penicillium chrysogenum, Pex14/17 is involved in the penicillin biosynthesis process (19). Most importantly, the loss of *AoPEX14/17* contributed to the accumulation of arthrobotrisins of *A. oligospora*. Arthrobotrisins, polyketide synthase-terpenoid synthase hybrid metabolites found in *A. oligospora* and other NT fungi, have been shown to inhibit hyphal growth and trap formation (35, 45). Fungal hyphal growth and pathogenicity were inhibited in Δ*Aopex14/17* mutants associated with the upregulated arthrobotrisin content. The mechanism underlying the ability of Pex14/17 to regulate these processes in *A. oligospora* awaits further analysis.

AoPex14, AoPex13, and AoPex14/17 are the components of peroxisomal docking complex, and we demonstrated by Y2H that AoPex14/17 interacted with AoPex14 but not with AoPex13. A previous study found that MoPex14 interacted with MoPex13 and MoPex14/17, but MoPex13 did not bind to MoPex14/17 in M. oryzae (46), which is consistent with our results. The Y2H data support the conclusion that AoPex14 binds primarily to AoPex14/17 and is essential for the maintenance of AoPex14/17 on the peroxisomal membrane.

In our recent work, AoPex1 and AoPex6 are required for mycelial growth, trap formation, and conidiation of *A. oligospora* (29), and the Δ*Aopex1* and Δ*Aopex6* mutants showed a more significant phenotypic difference than the Δ*Aopex14/17* mutant, such as mycelial growth and trap formation. Pex1 and Pex6 are both cytosolic and membrane-associated AAA ATPases of the peroxisomal protein import machinery, which have been demonstrated to mediate the ATP-dependent dislocation of the PTS1 receptor from the peroxisomal membrane to the cytosol (47); while Pex14/17 is a docking complex component specific to filamentous fungi and is mainly involved in the docking of receptors and cargoes during peroxisome synthesis (21). Thus, Pex1 and Pex6 are functionally different from Pex14/17, which would result in their phenotypic differences in *A. oligospora*.

**FIG 8:**
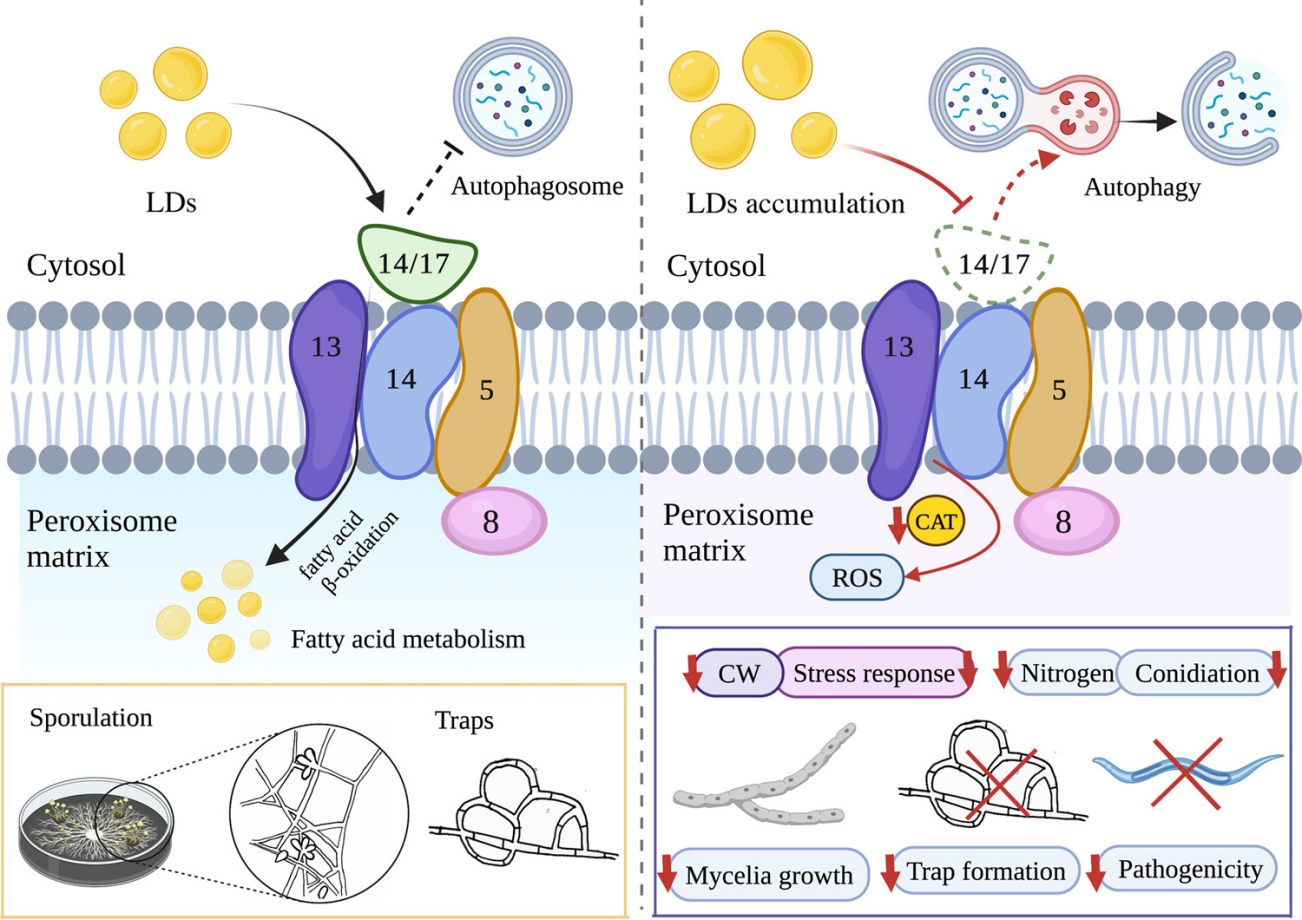
Schematic illustration of the regulation of *AoPEX14/17* in *A. oligospora*. In *A. oligospora*, the deletion of *AoPEX14/17* inhibited the fatty acid metabolism and resulted in reduced CAT activity and increased LDs accumulation and ROS level; meanwhile, the absence of *AoPEX14/17* impaired the autophagy, stress response, and endocytosis. These combined effects resulted in a reduction in mycelial growth, sporulation, trap formation, and pathogenicity. ROS, reactive oxygen species. CAT, catalase. LDs, lipid droplets. CW, cell wall.

In conclusion, we characterized the function of Pex14/17 in *A. oligospora* involved in mycelial growth and development, conidial production, pathogenicity, lipid utilization, lipid metabolism, response to oxidative stress, ROS accumulation, autophagy, endocytosis, the maintenance of structure and function of organelles, and the regulation of secondary metabolites (Fig. 8). These findings provide a basis for exploring the features of peroxins that underlie the survival and environmental adaptations of NT fungi and contribute to developing an effective biocontrol strategy for pathogenic nematodes.

## MATERIALS AND METHODS

### Strains, media, and growth conditions.

*Arthrobotrys oligospora* ATCC 24927 and its derived mutants were grown on potato dextrose agar (PDA) medium in a constant-temperature incubator (28°C). The plasmid pCSN44 containing the selective marker hygromycin resistance gene (*hph*) was stored in the Escherichia coli strain DH5α (TaKaRa, Shiga, Japan), and the knockout vector was created using the plasmid PRS426 (48). As previously mentioned, S. cerevisiae (FY834) was cultivated in yeast extract peptone dextrose (YPD) medium for the recombinational cloning technique (49). To investigate mycelial development and associated phenotypic characteristics, the following media were used: TYGA (10 g tryptone, 5 g yeast extract, 10 g glucose, and 5 g molasses per 1 L), TG (10 g tryptone and 10 g glucose per 1 L), and CMY (20 g maizena [corn starch] and 5 g yeast extract per 1 L); additionally, 20 g agar should be added per 1 L of the above media (50). In addition, protoplast regeneration was conducted with PDAS (200 g potato, 0.3 g yeast extract, 0.6 M sucrose, and 10 g molasses per 1 L) medium. The nematode Caenorhabditis elegans was cultured in WA (20 g agar per 1 L) medium. Besides this, the utilization of carbon sources was also tested in MM (20 g glucose, 2 g NaNO_3_, 0.01 g FeSO_4_·7H_2_O, and 20 g agar per 1 L) medium.

### Sequence and phylogenetic analyses of *AoPex14/17*.

The amino acid sequences for Pex14/17 homologs were obtained from the genomic databases of the model fungi S. cerevisiae, N. crassa, and A. nidulans, and the orthologous AoPex14/17 (AOL_s00081g263) was identified in *A. oligospora*. The NCBI online website (http://www.ncbi.nlm.nih.gov/) was used to download all protein sequences. The sequence similarity of homologous Pex14/17 from diverse fungi was analyzed by DNAman software (Lynnon Biosoft, San Ramon, USA). ClustalW was used to perform multiple sequence alignments, Mega X was used to construct a neighbor-joining tree, and the analysis of bootstrap values was conducted using 1,000 replicates (51).

### Targeted gene deletion and Southern blotting.

Using the homologous recombination technique, the *AoPEX14/17* gene was deleted in the wild-type (WT) strain (52). The upstream and downstream fragments of *AoPEX14/17* were PCR amplified from *A. oligospora* using a modified yeast cloning procedure, and a hygromycin cassette (*hph*) was amplified using the vector pCSN44 as a template (53). The fragment, *hph* cassette, and gapped yeast shuttle vector pRS426 (digested with EcoRI and XhoI) were transformed into yeast strain FY834. Next, the recombinant cloned strains were screened by SC-Ura medium as described previously (53, 54). The circular construct was created by homologous recombination, and the disruption vector (pRS426-*AoPEX14/17*-*hph*) was eventually recovered through transformation into E. coli DH5α. The gene disruption target fragment was amplified using paired primers (see Table S3 in the supplemental material) and transformed into *A. oligospora* protoplasts. As previously mentioned, hygromycin-resistant transformants were selected on PDAS containing 200 μg/mL hygromycin (55). The candidates for the gene deletion mutant were screened from hygromycin-resistant transformants via genomic PCR and confirmed by Southern blotting.

10.1128/msphere.00012-23.10 TABLE S3 List of primers used in this study. Download Table S3, DOCX file, 0.02 MB. Copyright © 2023 Liu et al. 2023 Liu et al. https://creativecommons.org/licenses/by/4.0/ This content is distributed under the terms of the Creative Commons Attribution 4.0 International license.

Genomic DNA was digested with NcoI enzyme (New England Biolabs, Inc., Ipswich, MA, USA) for Southern blotting. The analysis was carried out in accordance with the directions included in the North2South chemiluminescent hybridization and detection kit (Pierce, Rockford, IL, USA).

### RNA sequencing analysis.

WT and mutant strains were incubated in PD (200 g potatoes and 20 g glucose per 1 L) broth medium at 28°C for 5 and 7 days, respectively. Three replicates were used for each sample. Mycelial samples were collected and frozen in liquid nitrogen, followed by sequencing by Majorbio Bio-pharm Technology Co. Ltd (Shanghai, China). Then, the Majorbio Cloud platform was used to evaluate the data (www.majorbio.com). RNA was extracted using the AxyPrep Multisource RNA miniprep kit (Axygen, Jiangsu, China), transcriptome results were verified by RT-qPCR, and the selected genes and relative primers are listed in Table S3 (23, 24). Finally, differentially expressed genes (DEGs) were screened out using *P* ≤ 0.05 and Log_2_ (fold change) ≥ 2 as thresholds. The GO and KEGG databases were used to functionally assess the selected DEGs (56).

### Comparison of mycelial growth and conidia yield.

To determine mycelial growth, WT and mutant strains were inoculated onto TG, PDA, and TYGA media at 28°C for 5 days, respectively, with growth rates and colony diameters measured at 24-h intervals (57). To analyze sporulation, conidia were collected from 14-day CMY agar plates with 5 mL sterile distilled water. Conidia produced by a given colony were counted and reported as the total number of conidia present per unit area of the colony using a hemocytometer (58).

### Trap formation and bioassay.

To observe trap formation, 50-μL conidia suspensions (4 × 10^6^ conidia mL^−1^) of WT and mutant strains were evenly distributed on WA plates at 28°C for 3 to 4 days. Then, to induce trap formation, approximately 300 to 400 nematodes (C. elegans) were added to each WA plate (59). The number of traps produced and nematodes captured by each strain were counted using a microscope (Olympus, Japan) at 12, 24, 36, and 48 h.

### Analysis of stress tolerance.

To test the sensitivity of each strain to various environmental stresses, mycelial plugs taken from the periphery of each strain were inoculated on TG medium amended with the following compounds: menadione (0.01, 0.02, and 0.03 mM) and H_2_O_2_ (2.5, 5, and 10 mM) as oxidative stressors; Congo red (CR) (0.05, 0.07, and 0.1 mg/mL) and sodium dodecyl sulfate (SDS) (0.01, 0.02, and 0.03%) as cell wall stress agents; and NaCl (0.1, 0.2, and 0.3 M) and sorbitol (0.25, 0.5, and 0.75 M) as osmotic stressors. After incubation at 28°C for 6 days, the colony diameter in each plate was measured, and the relative growth inhibition (RGI) of each colony was evaluated as described previously (60). Each experiment was repeated three times.

### Analysis of fatty acid utilization.

For the lipid utilization assay, WT and mutant strains were inoculated on MM medium supplemented with sodium acetate (50 mM), oleic acid (0.12%), or Tween 20 (0.5%) as the sole carbon source. The size of the fungal colonies was measured and photographed postincubation at 28°C for 6 days, after which RGI values were determined (29).

### Analyses of reactive oxygen species and endocytosis.

By staining with nitrotetrazolium blue chloride (NBT) (Solarbio, Beijing, China), the ROS levels in the hyphae were determined. WT and mutant strains were grown on PDA at 28°C for 3 days, after which plates were stained with 20 mL of a 0.2% NBT solution and incubated in the dark at 28°C for 30 min. After draining the supernatant, ethanol was used to wash the plates. The plates were reincubated in the dark at 28°C for 30 min before imaging (61).

Cellular ROS accumulation in the fungal hyphae was determined by dihydroethidium (DHE) (Beyotime, Shanghai, China) staining. Briefly, the collected mycelia were mixed with 10 μg/mL DHE solution, incubated at 37°C for 30 min, and then washed with phosphate-buffered saline (PBS) twice (62). Subsequently, ROS levels were estimated from the intensity of DHE fluorescence with the same weight of mycelium via multimode microplate reader (Molecular Devices, Shanghai, China).

### Fluorescence microscopy and transmission electron microscope.

The fungal cell wall and hyphal septum were visualized by staining with 20 μg/mL calcofluor white (CFW) (Sigma-Aldrich, St. Louis, MO, USA), and mycelial cell nuclei were visualized by staining with 20 μg/mL 4′,6-diamidino-2-phenylindole (DAPI) and 20 μg/mL CFW (63). As previously described, lipid droplets (LDs) were stained with 10 μg/mL boron dipyrromethene (BODIPY) (Thermo Fisher Scientific, Waltham, MA, USA) (29). In addition, autophagy of the hyphae was detected by 100 μg/mL monodansylcadaverine (MDC) staining.

For TEM analysis, WT and mutant strains were harvested from colonies cultured on PD broth for 3 days. Then, the mycelia were collected and fixed with 2.5% glutaraldehyde for TEM observation (30).

### Reverse transcription-quantitative PCR.

Total RNA was extracted using TRIzol reagent (Invitrogen, Carlsbad, CA, USA) from frozen fungal tissues. Then, the treated RNA samples were reversely transcribed into cDNA using the PrimeScript RT reagent kit (TaKaRa, Shiga, Japan). The above experiments were performed according to the manufacturer's instructions. RT-qPCR was accomplished by LightCycler 480 with SYBR green I master mix (Roche, Basel, Switzerland) and a specific primer set (Table S3). Besides this, β-tubulin (AOL_s00076g640) was used as an internal control to normalize the target gene’s expression level (64). Finally, the relative expression level of duplicated samples was analyzed by the 2^−ΔΔ^*^CT^* method, and each reaction was independently repeated three times with three biological replicates per sample.

### Untargeted metabolomics analysis.

WT and mutant strains were inoculated in 250 mL of PD broth at 28°C for 5 days. Then, the mycelium was dissolved in 250 mL ethyl acetate, and the fermentation broth was ultrasonically extracted and allowed to stand overnight. The ethyl acetate layer was dried with a rotary evaporator before being dissolved in 1 mL of chromatographic methanol. For liquid chromatography-mass spectrometry (LC-MS) analysis, the methanol solution was filtered through a 0.22-μm membrane filter. Analysis was performed using the Thermo Fisher Scientific Dionex Ultimate 3000 UHPLC system and a Thermo Fisher high-resolution Q precision focusing mass spectrometer (Thermo Fisher Scientific) (30, 65, 66). Subsequently, the Compound Discoverer 3.3 software package was used to perform metabolite deconvolutions and identifications (66, 67).

### Y2H assay.

The cDNAs of *Aopex14/17* were cloned into bait plasmid pGBKT7 (BD-Pex14/17), and cDNAs of *Aopex13* and *Aopex14* were cloned into prey plasmid pGADT7 (AD-Pex13 and AD-Pex14). This protocol was experimented and analyzed according to previously described studies (60).

### Statistical analysis.

GraphPad Prism (version 9.00) was used for statistical analysis, and Tukey's multiple comparison test was applied to assess the level of difference between different strains. Data are presented as means ± standard deviation, and a *P* value of <0.05 was used to determine significant differences.

### Data availability.

All data generated or analyzed during this study are included in the published paper and associated supplemental files. The sequencing data were deposited in the Gene Expression Omnibus (GEO) (https://www.ncbi.nlm.nih.gov/geo/) under accession number GSE216978.
