# Supplementary figures and images for: Peroxin Pex14/17 Is Required for Trap Formation, and Plays Pleiotropic Roles in Mycelial Development, Stress Response, and Secondary Metabolism in Arthrobotrys oligospora
Source: mSphere. 2023 Feb 14;8(2):e00012-23. doi: 10.1128/msphere.00012-23 (PMC10117088; doi:10.1128/msphere.00012-23)

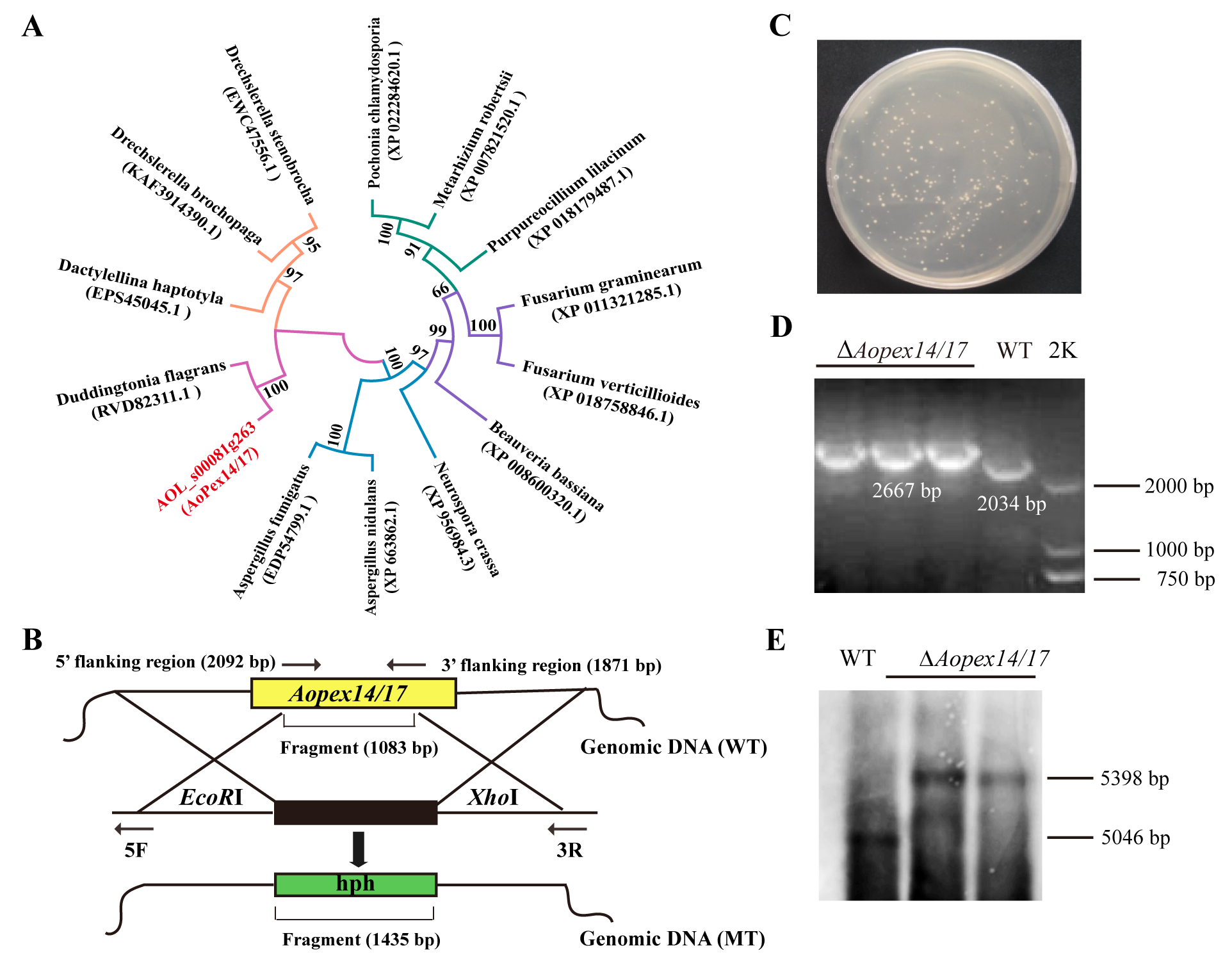

Supplement: FIG S1 [file msphere.00012-23-s0001.tif]

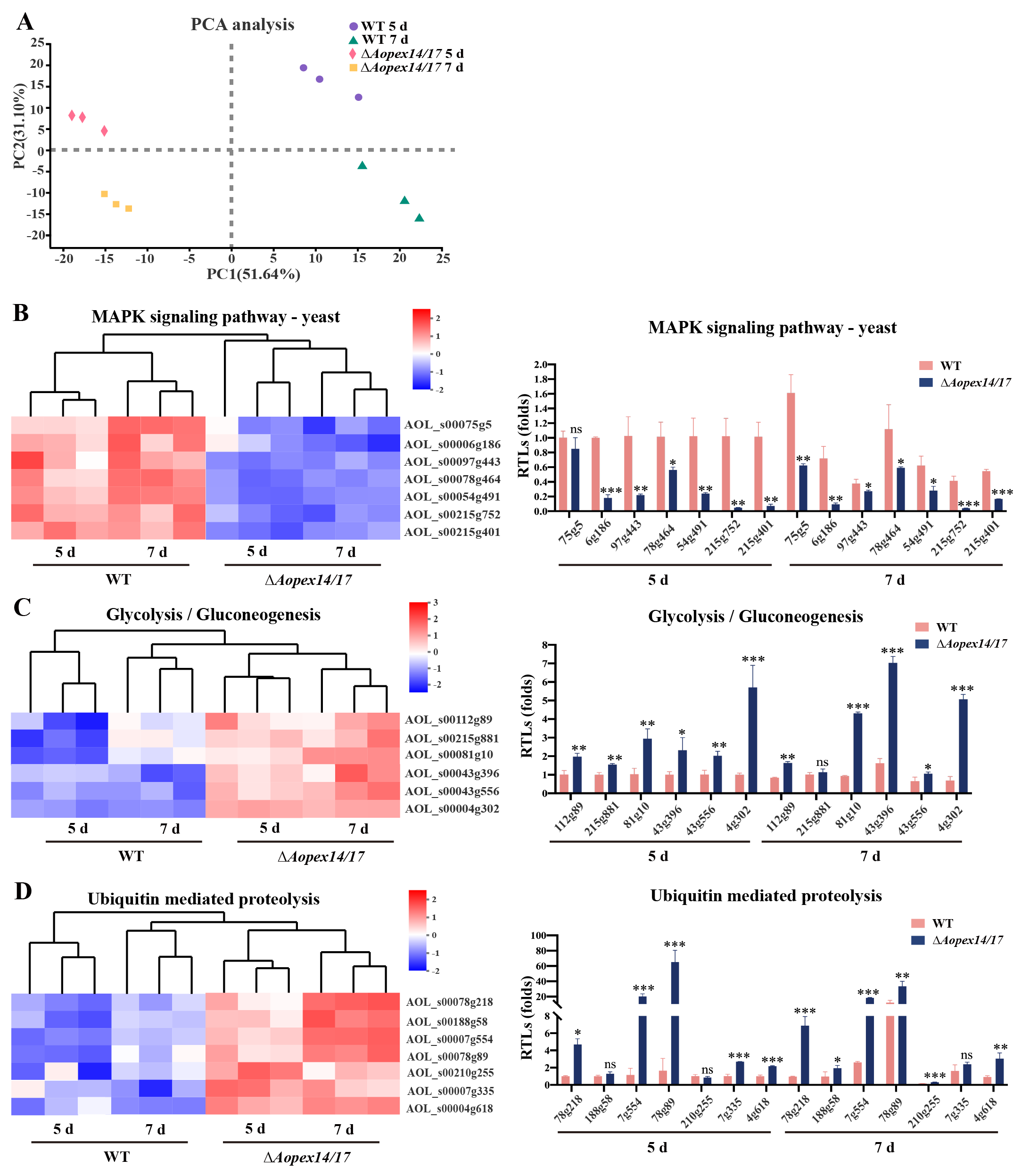

Supplement: FIG S2 [file msphere.00012-23-s0002.tif]

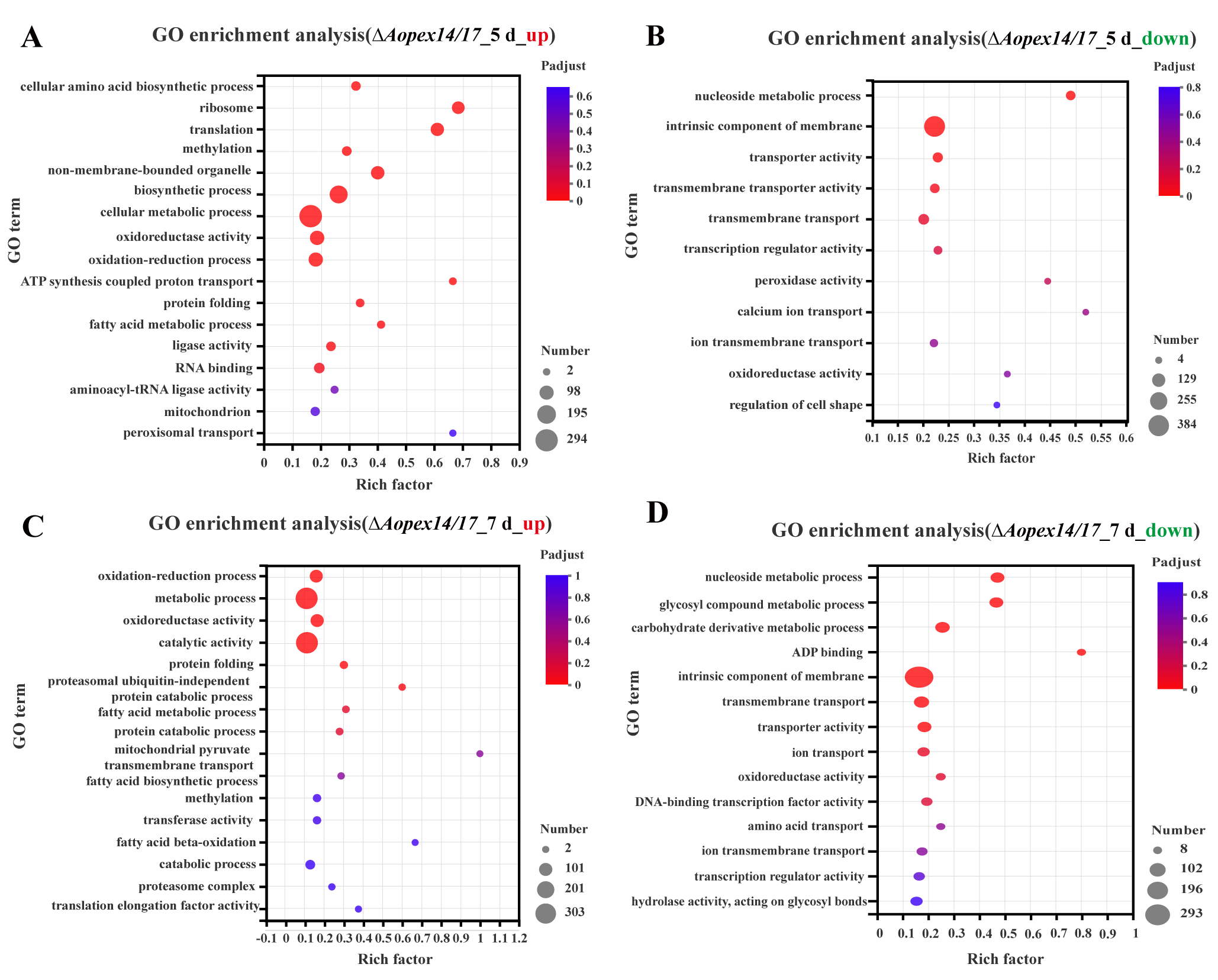

Supplement: FIG S3 [file msphere.00012-23-s0003.tif]

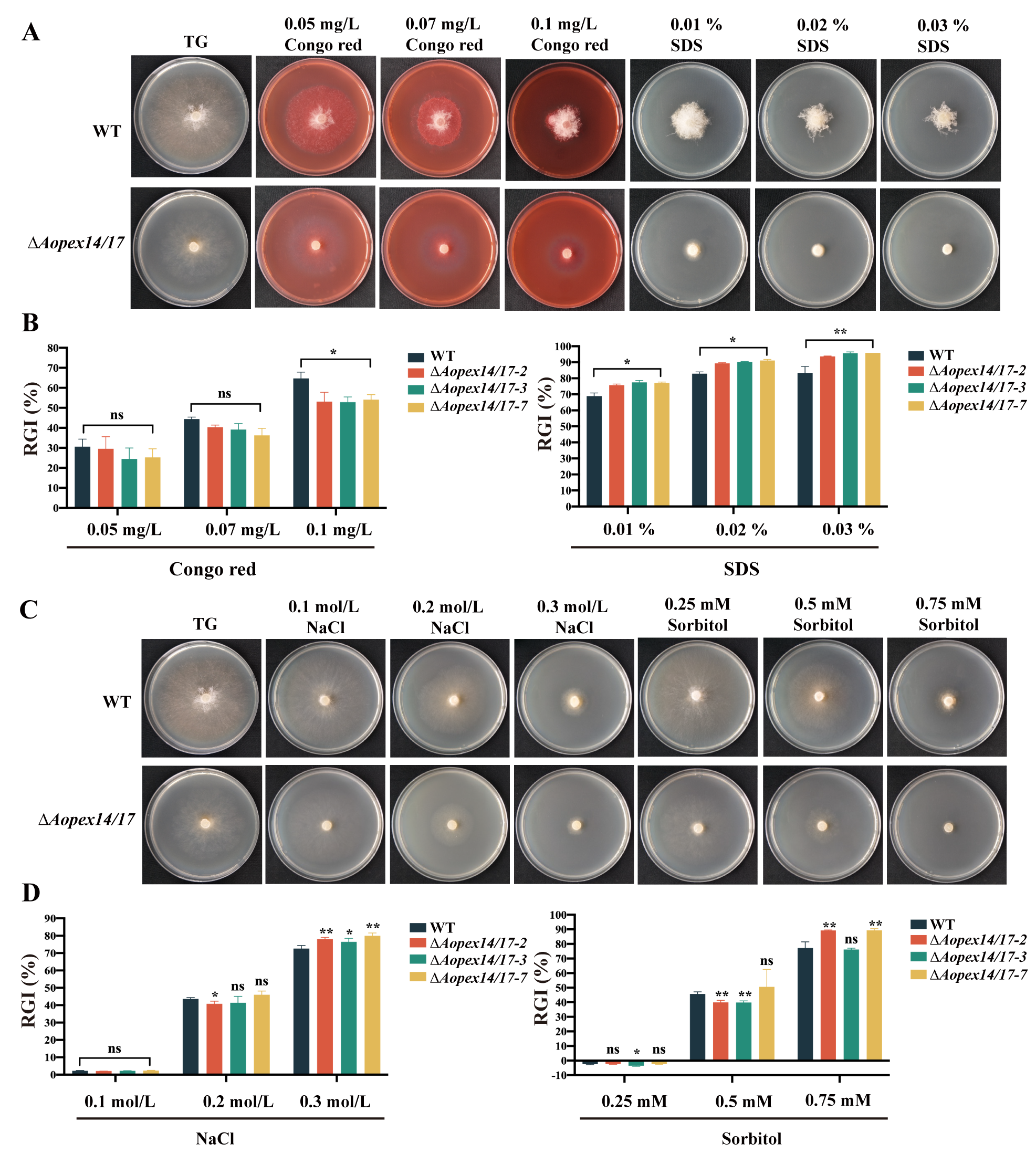

Supplement: FIG S4 [file msphere.00012-23-s0004.tif]

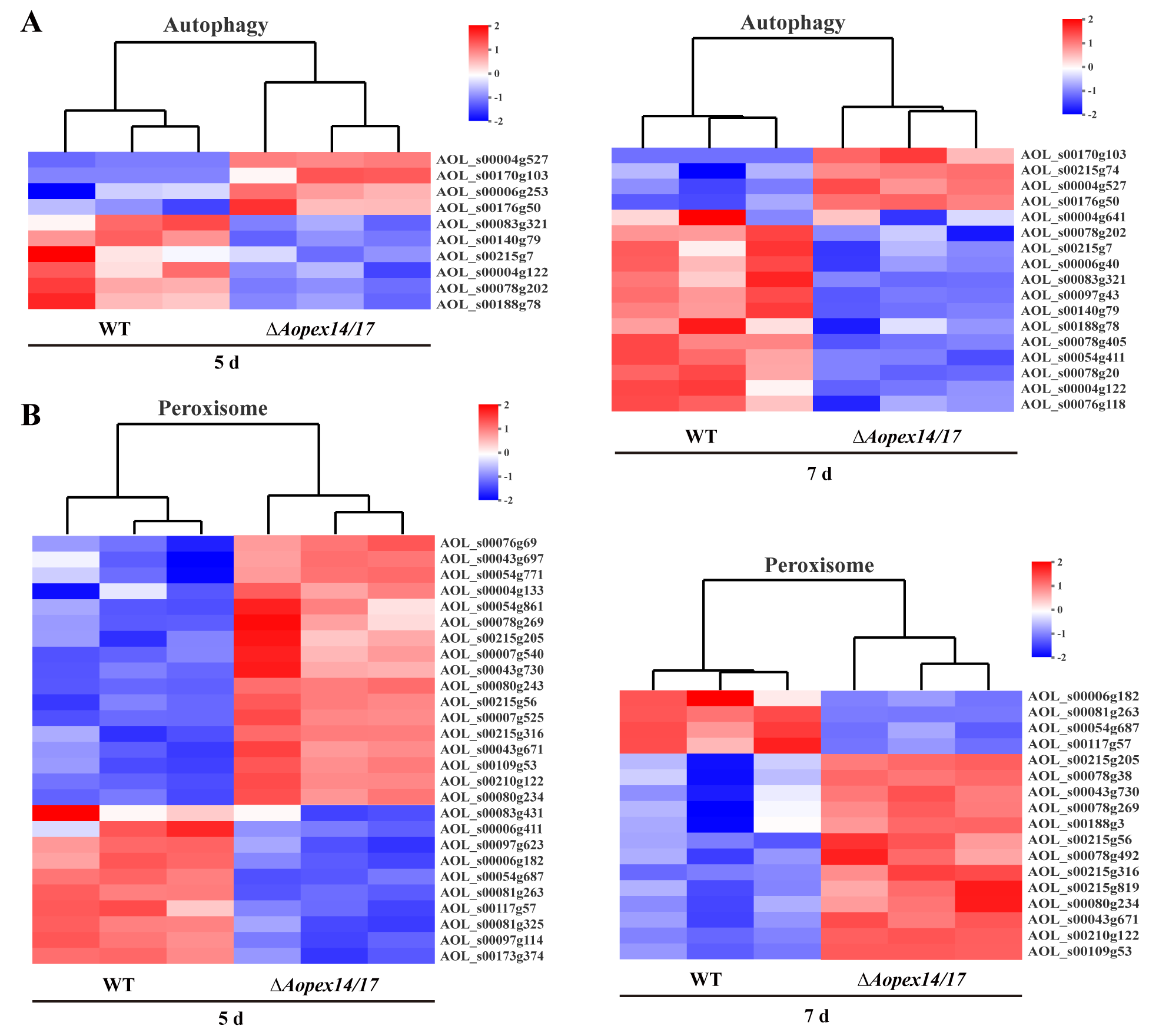

Supplement: FIG S5 [file msphere.00012-23-s0005.tif]

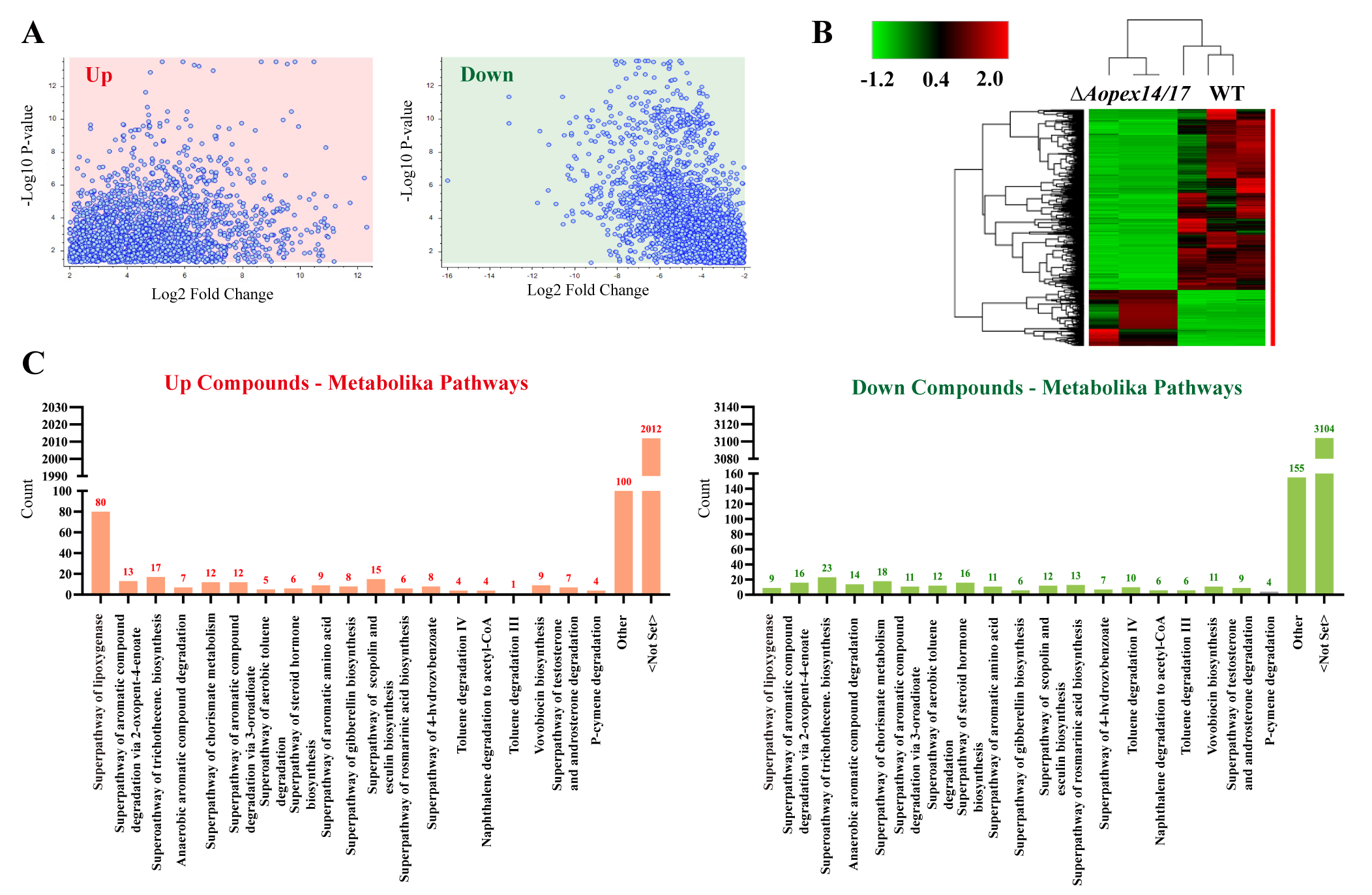

Supplement: FIG S6 [file msphere.00012-23-s0006.tif]

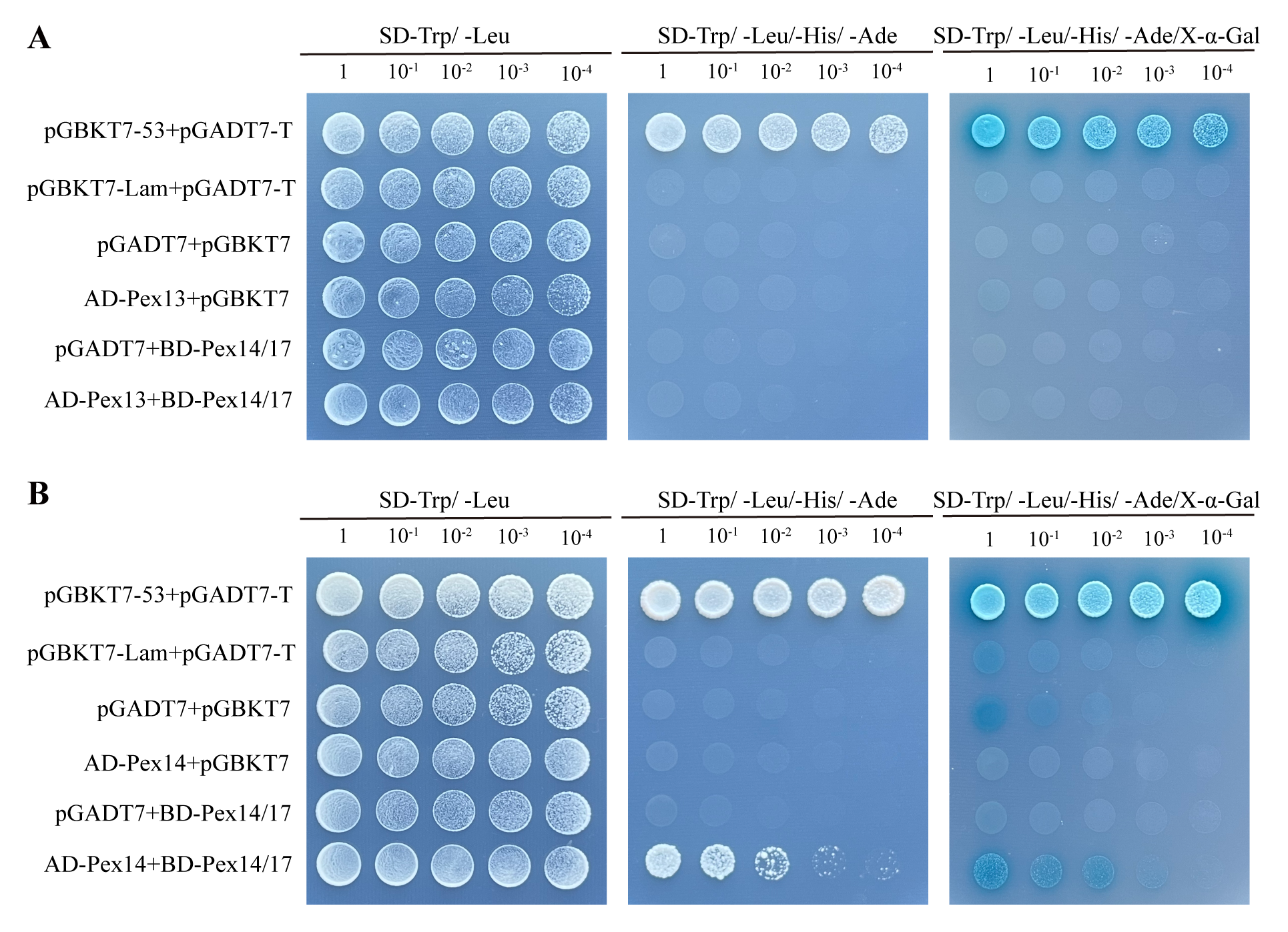

Supplement: FIG S7 [file msphere.00012-23-s0007.tif]
