# Supplementary material for: Peroxin Pex14/17 Is Required for Trap Formation, and Plays Pleiotropic Roles in Mycelial Development, Stress Response, and Secondary Metabolism in Arthrobotrys oligospora
Source: mSphere. 2023 Feb 14;8(2):e00012-23. doi: 10.1128/msphere.00012-23 (PMC10117088; doi:10.1128/msphere.00012-23)
Supplement: TABLE S1 [file msphere.00012-23-s0008.docx]

**Table S1**

| **Sample** | | **Total reads** | | **Total mapped** | | | **Multiple mapped** | | | **Uniquely mapped** | |
| --- | --- | --- | --- | --- | --- | --- | --- | --- | --- | --- | --- |
| A_5d_1 | | 56168652 | | 53984633(96.11%) | | | 263752(0.47%) | | | 53720881(95.64%) | |
| A_5d_2 | | 58278800 | | 56144119(96.34%) | | | 280976(0.48%) | | | 55863143(95.85%) | |
| A_5d_3 | | 47717600 | | 45879186(96.15%) | | | 221332(0.46%) | | | 45657854(95.68%) | |
| A_7d_1 | | 42953858 | | 41199163(95.91%) | | | 158373(0.37%) | | | 41040790(95.55%) | |
| A_7d_2 | | 42587644 | | 41039204(96.36%) | | | 156144(0.37%) | | | 40883060(96.0%) | |
| A_7d_3 | | 51242914 | | 49321224(96.25%) | | | 200843(0.39%) | | | 49120381(95.86%) | |
| H_5d_1 | | 43065058 | | 41777856(97.01%) | | | 187859(0.44%) | | | 41589997(96.57%) | |
| H_5d_2 | | 51066508 | | 49138854(96.23%) | | | 309701(0.61%) | | | 48829153(95.62%) | |
| H_5d_3 | | 47843426 | | 46162016(96.49%) | | | 282316(0.59%) | | | 45879700(95.9%) | |
| H_7d_1 | | 52942360 | | 50769655(95.9%) | | | 268482(0.51%) | | | 50501173(95.39%) | |
| H_7d_2 | | 55860766 | | 53575968(95.91%) | | | 249980(0.45%) | | | 53325988(95.46%) | |
| H_7d_3 | | 54883710 | | 52872170(96.33%) | | | 230179(0.42%) | | | 52641991(95.92%) | |
| **Sample** | **Raw reads** | | **Raw bases** | | **Clean reads** | **Clean bases** | | **Error rate(%)** | **Q20(%)** | **Q30(%)** | **GC content(%)** |
| A_5d_1 | 56630662 | | 8551229962 | | 56168652 | 8290833502 | | 0.0254 | 97.83 | 93.85 | 48.03 |
| A_5d_2 | 58759646 | | 8872706546 | | 58278800 | 8563239385 | | 0.0255 | 97.77 | 93.69 | 47.79 |
| A_5d_3 | 48092042 | | 7261898342 | | 47717600 | 7062196705 | | 0.0255 | 97.78 | 93.72 | 47.57 |
| A_7d_1 | 43330726 | | 6542939626 | | 42953858 | 6338021606 | | 0.0256 | 97.72 | 93.62 | 48.06 |
| A_7d_2 | 42911438 | | 6479627138 | | 42587644 | 6297627480 | | 0.0254 | 97.82 | 93.76 | 48.09 |
| A_7d_3 | 51606800 | | 7792626800 | | 51242914 | 7553457355 | | 0.0252 | 97.89 | 93.98 | 48.21 |
| H_5d_1 | 43451856 | | 6561230256 | | 43065058 | 6298610542 | | 0.0252 | 97.91 | 94.02 | 47.93 |
| H_5d_2 | 51519808 | | 7779491008 | | 51066508 | 7534388399 | | 0.0253 | 97.85 | 93.92 | 48.01 |
| H_5d_3 | 48220928 | | 7281360128 | | 47843426 | 7094148816 | | 0.0252 | 97.91 | 94.03 | 48.17 |
| H_7d_1 | 53343186 | | 8054821086 | | 52942360 | 7776908006 | | 0.0252 | 97.9 | 94.01 | 47.95 |
| H_7d_2 | 56427986 | | 8520625886 | | 55860766 | 8081634032 | | 0.0254 | 97.79 | 93.82 | 48.09 |
| H_7d_3 | 55353040 | | 8358309040 | | 54883710 | 8002022092 | | 0.0254 | 97.81 | 93.8 | 48.09 |
