# Supplementary material for: Peroxin Pex14/17 Is Required for Trap Formation, and Plays Pleiotropic Roles in Mycelial Development, Stress Response, and Secondary Metabolism in Arthrobotrys oligospora
Source: mSphere. 2023 Feb 14;8(2):e00012-23. doi: 10.1128/msphere.00012-23 (PMC10117088; doi:10.1128/msphere.00012-23)
Supplement: TABLE S2 [file msphere.00012-23-s0009.docx]

**Table S2**

| **Name**  **(The top twenty compounds with significant changes)** | | **Formula** | **Molecular**  **Weight** | **RT**  **[min]** | **Log2 Fold**  **Change:**  **Δ*Aopex14/17*/WT** | | **P-value:**  **Δ*Aopex14/17*/WT** | |
| --- | --- | --- | --- | --- | --- | --- | --- | --- |
| (+/-)9-HpODE | | C_18_H_32_O_4_ | 312.23025 | 32.004 | 4.99 | | 0.025895917 | |
| ethyl 1-[4-({[4-(tert-butyl)anilino]carbonyl}amino)phenyl]-3,5-dimethyl-1H-pyrazole-4-carboxylate | | C_25_H_30_N_4_O_3_ | 434.22995 | 28.554 | -1.35 | | 0.564088352 | |
| NP-020403 | | C_17_ H_20_ O_3_ | 254.13035 | 27.663 | -0.33 | | 0.811531458 | |
| (2,6-Dimethylfuro[2,3-f][1]benzofuran-3,7-diyl)bis[(4-methyl-1-piperazinyl)methanone] | | C_24_H_30_N_4_O_4_ | 438.22539 | 23.003 | -2.65 | | 0.373206183 | |
| (2,6-Dimethylfuro[2,3-f][1]benzofuran-3,7-diyl)bis[(4-methyl-1-piperazinyl)methanone] | | C_24_H_30_N_4_O_4_ | 438.22537 | 22.727 | -3.59 | | 0.159665085 | |
| octadecatetraenoic acid | | C_18_H_28_O_2_ | 276.20871 | 31.992 | 5.97 | | 0.002361478 | |
| NP-017061 | | C_20_ H_30_ O_4_ | 356.19831 | 22.938 | -0.97 | | 0.634593329 | |
| 13(S)-HOTrE | | C_18_H_30_O_3_ | 294.21926 | 34.325 | 5.4 | | 0.043756298 | |
| 12-Oxo phytodienoic acid | | C_18_H_28_O_3_ | 274.1931 | 33.524 | 5.03 | | 0.000759163 | |
| 3-[4-(2-{[(4,4-Dimethyl-2,6-dioxocyclohexylidene)methyl]amino}ethyl)-1-piperazinyl]-1-(3-methoxyphenyl)-2,5-pyrrolidinedione | | C_26_H_34_N_4_O_5_ | 482.25166 | 31.576 | -1.75 | | 0.586819577 | |
| 1-(4-Hexyloxy-phenyl)-3-(4-pyridin-2-yl-piperazin-1-yl)-pyrrolidine-2,5-dione | | C_25_H_32_N_4_O_3_ | 436.24625 | 31.57 | -1.33 | | 0.664336364 | |
| NP-001445 | | C_18_H_30_O_5_ | 308.1985 | 26.144 | 9.08 | | 0.00115367 | |
| 12-Oxo phytodienoic acid | | C_18_H_28_O_3_ | 292.20359 | 18.938 | 5.33 | | 0.424547071 | |
| NP-016928 | | C_20_H_28_O_3_ | 338.18769 | 22.923 | -1.06 | | 0.603821084 | |
| 19-Norandrostenedione | | C_18_ H_24_O_2_ | 272.17737 | 26.137 | 8.78 | | 0.001304752 | |
| ML-236C | | C_18_H_26_O_3_ | 290.18789 | 26.135 | 10.87 | | 0.000403604 | |
| NP-016928 | | C_20_H_28_O_3_ | 338.18777 | 28.549 | -0.61 | | 0.740350743 | |
| NP-002322 | | C_18_H_32_O_4_ | 294.2193 | 29.852 | 7.83 | | 0.000179029 | |
| (+/-)9-HpODE | | C_18_H_32_O_4_ | 312.23029 | 29.862 | 7.95 | | 0.000279787 | |
| N-(1-adamantyl)-N'-(2,2-diethoxyethyl)thiourea | | C_17_H_30_N_2_O_2_S | 326.20942 | 26.13 | 10.65 | | 0.000419873 | |
|  | **Up-regulated Metabolic Pathways** | | | | | **Count** | | **Percent (%)** |
| 1 | Superpathway of lipoxygenase | | | | | 80 | | 3.42 |
| 2 | Superpathway of trichothecene biosynthesis | | | | | 17 | | 0.73 |
| 3 | Superpathway of scopolin and esculin biosynthesis | | | | | 15 | | 0.64 |
| 4 | Superpathway of aromatic compound degradation via 2-oxopent-4-enoate | | | | | 13 | | 0.56 |
| 5 | Superpathway of aromatic compound degradation via 3-oxoadipate | | | | | 12 | | 0.51 |
| 6 | Superpathway of chorismate metabolism | | | | | 12 | | 0.51 |
| 7 | Novobiocin biosynthesis | | | | | 9 | | 0.38 |
| 8 | Superpathway of 4-hydroxybenzoate biosynthesis (yeast) | | | | | 8 | | 0.34 |
| 9 | Superpathway of gibberellin biosynthesis | | | | | 8 | | 0.34 |
| 10 | Anaerobic aromatic compound degradation (Thauera aromatica) | | | | | 7 | | 0.3 |
|  | **Down-regulated Metabolic Pathways** | | | | | **Count** | | **Percent (%)** |
| 1 | Superpathway of aromatic compound degradation via 2-oxopent-4-enoate | | | | | 16 | | 0.46 |
| 2 | Superpathway of trichothecene biosynthesis | | | | | 23 | | 0.66 |
| 3 | Anaerobic aromatic compound degradation (Thauera aromatica) | | | | | 14 | | 0.4 |
| 4 | Superpathway of chorismate metabolism | | | | | 18 | | 0.52 |
| 5 | Superpathway of aromatic compound degradation via 3-oxoadipate | | | | | 11 | | 0.32 |
| 6 | Superpathway of aerobic toluene degradation | | | | | 12 | | 0.35 |
| 7 | Superpathway of steroid hormone biosynthesis | | | | | 16 | | 0.46 |
| 8 | Superpathway of aromatic amino acid biosynthesis | | | | | 11 | | 0.32 |
| 9 | Superpathway of rosmarinic acid biosynthesis | | | | | 13 | | 0.37 |
| 10 | Superpathway of scopolin and esculin biosynthesis | | | | | 12 | | 0.35 |
