# Supplementary material for: Peroxin Pex14/17 Is Required for Trap Formation, and Plays Pleiotropic Roles in Mycelial Development, Stress Response, and Secondary Metabolism in Arthrobotrys oligospora
Source: mSphere. 2023 Feb 14;8(2):e00012-23. doi: 10.1128/msphere.00012-23 (PMC10117088; doi:10.1128/msphere.00012-23)
Supplement: TABLE S3 [file msphere.00012-23-s0010.docx]

**Table S3**

| **Primers** | **Sequence (5′-3′)** | **Description** |
| --- | --- | --- |
| AoPEX14/17-5F | GTAACGCCAGGGTTTTCCCAGTCACGACGAACAGATGACGAGGAGTTTAGG | Amplify the *AoPEX14/17* gene 5’ flank |
| AoPEX14/17-5R | ATCCACTTAACGTTACTGAAATCTCCAACGGTGACGAAGATGAGTTTGG |  |
| AoPEX14/17-3F | CTCCTTCAATATCATCTTCTGTCTCCGACTTTGGACTTCGGATTCTT | Amplify the *AoPEX14/17* gene 3’ flank |
| AoPEX14/17-3R | GCGGATAACAATTTCACACAGGAAACAGCGTCAACTTCTACCTCACCCT |  |
| Hph-F | GTCGGAGACAGAAGATGATATTGAAGGAGC | Amplify the *hph* cassette |
| Hph-R | GTTGGAGATTTCAGTAACGTTAAGTGGAT |  |
| AoPEX14/17-YZR | GGTTGAAGGGTTAGAGTAGGAA | Verify the transformants |
| AoPEX14/17-YZF | AGAGCCACAATCCAGTCCAG |  |
| AoPEX14/17-TZF | TAGAGTCGGTGCTGGAGG | Make Southern blotting probe |
| AoPEX14/17-TZR | AAAGAACCCGTTCGTTGC |  |
| **Lipid metabolism genes** | **Sequence (5′-3′)** | **Sequence (3′-5′)** |
| AOL_s00004g288 | AAGAAATCCCACTTCAGAGAGG | TACGTGTCCAGTAACATAGCTC |
| AOL_s00081g51 | GCCGATCCTTACCAAATCATTC | CCAATTCTTTTCCGTAGCTGAG |
| AOL_s00043g424 | CTCTGCTCTATGGATACGAACA | AGATGAACTTCTCGACTTCTCC |
| AOL_s00210g122 | GCCGCACATATTGTTAACAGAT | TGATCTTGCTGTTCTCAGTCAT |
| AOL_s00110g113 | CTAACAGAAACTCAAGCATCGG | GGAACCGGATTCATGAAATGAG |
| AOL_s00079g276 | AACAATCCGTCGTTATTGTTCC | GCGATCATGTAGTCTAGTCCTC |
| AOL_s00054g29 | GGTATCTACGGAAATTTTGGCC | GTGCAATATAATCGGGCTTGAG |
| AOL_s00004g606 | TTCGGATTCGTTATTACCTCCC | TAACATGAGTCGCTTGTTTGTG |
| **Sporulation genes** | **Sequence (5′-3′)** | **Sequence (3′-5′)** |
| AOL_s00173g221 (*wetA*) | TTACATGCCACCCCAAGTCC | CAATTGCAACTGCGTCCACA |
| AOL_s00080g63 (*abaA*) | AACTTTATGCGCCTTGTCGT | TTGGCTAGGTGGTCTGTACG |
| AOL_s00075g211 (*nsdD*) | ATTACGGCCGCCTAGTAGTC | CTCGTTTGGACCTGGTTGTG |
| AOL_s00169g18 (*veA*) | AAGCTACACCCAATCAACGC | TTGCGATGCTGACGATCTTG |
| AOL_s00054g811 (*velB*) | ATTCCGCAACTTCTCCCTCA | GGCATGTTTGGATTCTGGGG |
| AOL_s00007g157 (*flbC*) | CTCTCCGGCAAAGACAATCG | CTCTCCGGCAAAGACAATCG |
| AOL_s00043g361 (*fluG*) | GATTCCAGTCCCGTGAATTC | GCTAAGGAGAGGATGGGCAT |
| AOL_s00097g514 (*brlA*) | TTGAGGCCTCGATCCGTAGA | AGGTAGATGGCGCTGTTACG |
| AOL_s00006g570 (*hyp1*) | GCGGATCCAACATGAAGCTT | GGTTGACAACTGGGATGCTG |
| **Oxidants-degrading genes** | **Sequence (5′-3′)** | **Sequence (3′-5′)** |
| AOL_s00054g13 (*glr*) | CGAAAAGTCTGAATCGGGTGA | GCTCCACTTTGCCACATACATC |
| AOL_s00169g61 (*glt*) | AGAAACCTACGACCCAAACCA | CCCTTCCACCTCCTGAACTT |
| AOL_s00076g248（*thi*） | CTACTCCTCTGAAGCTGCTC | TCCATAGCTGCCATCATCGT |
| AOL_s00173g374（*cat-1*） | TCCCCATCCTCATCCATACG | GATAGCGGGCATTTCTTTCC |
| AOL_s00215g326 (*per*) | CACCATCCGCTCTGTCTTCA | GACGGCATCCTCGGTCTTGA |
| AOL_s00188g243 (*cat-2*) | GGGAAAGTTCGTTTACATCAAG | GTAGCAAGCAGTCCAGGTCAC |
| **MAPK signaling pathway - yeast genes** | **Sequence (5′-3′)** | **Sequence (3′-5′)** |
| AOL_s00075g5 | CAATCTTCCGTCATACTTGCAG | CGTATCTGTTTGAATGAGGCTG |
| AOL_s00006g186 | CGAAATGGGTCTCCGATACTTA | CATTCTTCGTATGTAGGCTTGC |
| AOL_s00097g443 | CGAATATCGGGTGTCAGAGTTA | CAAACTCCAAATATCGCTCTGG |
| AOL_s00078g464 | GGTGTATTTGTTGGTGTAGCAG | TTATTACTGGTGCCTTGTCCTG |
| AOL_s00054g491 | CATGTTAATCGCAACTCTCTCG | AGAATTGAACAATGCCGAGAAC |
| AOL_s00215g752 | ACCGAAGCAGAACAAGTTTTAC | GGACCTAGTAGATACTTGCGAC |
| AOL_s00215g401 | CAGAGTGCAAAAGTGTCTTTGA | TGGTTATCTCCACTAGATGTGC |
| **Glycolysis / Gluconeogenesis genes** | **Sequence (5′-3′)** | **Sequence (3′-5′)** |
| AOL_s00112g89 | CAAGAAGGGTGTTCCAATCAAG | AGATACAACCGATCTTTGTCGT |
| AOL_s00215g881 | GATAAAATCGTTCCCGGTGATG | CAACATCCTTCTCACCAGTTTG |
| AOL_s00081g10 | CGTTGACTTCATGACTAGCAAG | CTAGCCTTTAGGAATGCGTTTG |
| AOL_s00043g396 | TCTGCCATACTGATCAGTACAC | ATTTGTCTTTCCACTCTTGCAG |
| AOL_s00043g556 | GGACGACCGAATGTTACTTTTG | TTTTCACAACCAAATACCACCG |
| AOL_s00004g302 | ATTCAAAAGATCGCACGTATCG | ATTACTAGCAGCAGGTGTACTC |
| **Ubiquitin mediated proteolysis genes** | **Sequence (5′-3′)** | **Sequence (3′-5′)** |
| AOL_s00078g218 | GAGCTCTAACCCTCTAGTGAAC | CGGGCACGCTTATAGACTTTAT |
| AOL_s00188g58 | GAAGGAAGACCGTATTTTGTCG | CCCATAGACACGAATGTACTGT |
| AOL_s00007g554 | GACTATCCCTTCAAACCTCCAA | GGTCTTGTAAACATGAGCGATC |
| AOL_s00078g89 | TTCCAACTTGAACCGTCAATTC | CCAACAAGGTTGACAAGTGTAG |
| AOL_s00210g255 | CAAGGTCACCTTCATCAACAAC | CTGTTCCTTAACACCACCTTTG |
| AOL_s00007g335 | AACTTCACCACCAAGATCTACC | CCTTGCTGTCTTTTCAAAGGAA |
| AOL_s00004g618 | CGATGAATGATCAGGATGCAAA | AATACATGAATTGCGTTACCGG |
| AOL_s00076g640 (*tub*) | CCACCTTCGTCGGTAACTC | TCGTCCATACCCTCACCAG |
